# Familial Hypercholesterolemia: New Horizons for Diagnosis and Effective Management

**DOI:** 10.3389/fphar.2018.00707

**Published:** 2018-07-12

**Authors:** Maria Mytilinaiou, Ioannis Kyrou, Mike Khan, Dimitris K. Grammatopoulos, Harpal S. Randeva

**Affiliations:** ^1^Warwickshire Institute for the Study of Diabetes, Endocrinology and Metabolism, University Hospitals Coventry and Warwickshire NHS Trust, Coventry, United Kingdom; ^2^Aston Medical Research Institute, Aston Medical School, Aston University, Birmingham, United Kingdom; ^3^Division of Translational and Experimental Medicine, Warwick Medical School, University of Warwick, Coventry, United Kingdom; ^4^Centre of Applied Biological and Exercise Sciences, Faculty of Health and Life Sciences, Coventry University, Coventry, United Kingdom; ^5^Institute of Precision Diagnostics and Translational Medicine, Coventry and Warwickshire Pathology Service, University Hospitals Coventry and Warwickshire NHS Trust, Coventry, United Kingdom

**Keywords:** familial hypercholesterolemia (FH), heterozygous FH (HeFH), homozygous FH (HoFH), atherosclerotic cardiovascular disease (ASCVD), low density lipoprotein (LDL), statins, proprotein convertase subtilisin/kexin type 9 inhibitors (PCSK9 inhibitors)

## Abstract

Familial hypercholesterolemia (FH) is a common genetic cause of premature cardiovascular disease (CVD). The reported prevalence rates for both heterozygous FH (HeFH) and homozygous FH (HoFH) vary significantly, and this can be attributed, at least in part, to the variable diagnostic criteria used across different populations. Due to lack of consistent data, new global registries and unified guidelines are being formed, which are expected to advance current knowledge and improve the care of FH patients. This review presents a comprehensive overview of the pathophysiology, epidemiology, manifestations, and pharmacological treatment of FH, whilst summarizing the up-to-date relevant recommendations and guidelines. Ongoing research in FH seems promising and novel therapies are expected to be introduced in clinical practice in order to compliment or even substitute current treatment options, aiming for better lipid-lowering effects, fewer side effects, and improved clinical outcomes.

## Introduction

During the past decades, significant advances have been made in the prevention and treatment of atherosclerotic cardiovascular disease (ASCVD). Chronic exposure to high circulating cholesterol levels is a key atherogenic risk factor which characterizes familial hypercholesterolemia (FH) (Ito and Watts, 2015; Catapano et al., 2016), a genetic disease closely related with premature ASCVD and high mortality rates (Ito and Watts, 2015).

FH is a genetic disorder of the lipoprotein metabolism and constitutes one of the most common inherited metabolic disorders (Goldberg et al., 2011; Vickery et al., 2014). The underlying cause of FH is a genetic defect either of the low density lipoprotein receptor (LDLR) or of the proteins that regulate its metabolism, resulting in abnormally low uptake of low density lipoprotein (LDL) by the liver. Consequently, this leads to cholesterol accumulation in the circulation, which explains the associated high CVD risk (Hartgers et al., 2015; Ito and Watts, 2015; Baum et al., 2016). Of note, distinction between the maternal or paternal type of inheritance does not have an impact on the FH phenotype (Narverud et al., 2015).

FH presents with significant heterogeneity, depending on the specific gene defect and the variable degree of the accompanying high plasma LDL levels (Hovingh et al., 2013). As such, inheritance of only one mutant allele results in heterozygous familial hypercholesterolemia (HeFH), with reduced LDL clearance (2/3 of the normal rate) which leads to a 2- to 3-fold increase in circulating LDL (5–10 mmol/l; 200–400 mg/dl) (Parihar et al., 2012; Hovingh et al., 2013). The phenotypic expression of HeFH is particularly distributed between the third and sixth decade of life (Parihar et al., 2012). On the other hand, a genotype with both mutant alleles, either with the same (true homozygosity) mutation (pathogenic variant) or with different pathogenic variants (compound heterozygosity), translates to total absence or total defect of the LDLR (Ito and Watts, 2015). This leads to worse prognosis due to diminished LDLR functionality (European Association for Cardiovascular Prevention and Rehabilitation et al., 2011; Bouhairie and Goldberg, 2015), which depends crucially upon the levels of circulating LDL and not on the specific mutation (Hovingh et al., 2013).

Notably, in homozygous familial hypercholesterolemia (HoFH) patients the LDL clearance reaches only 1/3 of the normal rate according to previously published data, resulting in exponentially increased LDL plasma levels (Parihar et al., 2012). Thus, HoFH results in markedly high cholesterol levels (3- to 6-fold higher than normal; >15.5 mmol/l; >600 mg/dl; Hovingh et al., 2013; Ito and Watts, 2015). This leads to CVD due to atherosclerotic plaques and stenosis [e.g., coronary artery disease (CAD), calcifications in the aortic root and ascending aorta, aortic regurgitation, and even CVD death] usually first presenting during the first two decades of life (Kolansky et al., 2008; Hovingh et al., 2013; Ito and Watts, 2015; Raal et al., 2016b). After assessing and following up 39 HoFH patients, among whom there were 22 children ≤ 16 y.o., Kolansky et al. (2008) highlighted the presence of CVD even in the first decade of their lives and due to the progression of their CVD pathology in their teenage years, it is suggested that CVD risk screening could start early in childhood (Kolansky et al., 2008). It is important to also mention that more recent data present the great phenotypic heterogeneity regarding LDL levels (Foody and Vishwanath, 2016; Sanchez-Hernandez et al., 2016) and ASCVD, according to the type of the pathogenic variant in HoFH (e.g., true homozygotes vs. compound heterozygotes, gene involved, and null vs. defective alleles), hence suggesting that LDL clearance is probably related to the specific genotype (Sanchez-Hernandez et al., 2016).

This genetic derangement of the normal lipidemic/metabolic profile is well-known to induce atherogenesis, and, consequently, premature CVD (European Association for Cardiovascular Prevention and Rehabilitation et al., 2011; Nordestgaard et al., 2013). Not surprisingly, a significant proportion of the CVD events before the age of 45 is attributed to FH (Bouhairie and Goldberg, 2015). Indeed, the estimated risk of premature CVD in FH patients is 20-fold higher compared to that of the general population (Hovingh et al., 2013), and the lifetime CVD events are 3.9 times more likely than for patients with similar risk factors without FH (Villa et al., 2017). Of note, a recent multicenter study in Japanese patients (an ethnic population with low CVD incidence) reported that the prevalence of definite HeFH was 5.7% in patients with acute coronary syndrome (Ohmura et al., 2017). It should be also highlighted that such CAD events tend to present even sooner and most often in male patients (Neil et al., 2008). Overall, FH has been particularly associated with myocardial infarction (MI), angina pectoris, as well as peripheral arterial disease (PAD) and, hence, with increased mortality and disability-adjusted life years (Hutter et al., 2004; European Association for Cardiovascular Prevention and Rehabilitation et al., 2011; Nordestgaard et al., 2013; Perez de Isla et al., 2016).

Apart from the LDLR, other molecules like members of the scavenger receptor superfamily (SR-A1, SR-B1, SR-D1, SRE1, SR-F1, SR-H1&2) and the Lectin-like oxLDL receptor-1 (LOX1) are implicated in atherosclerosis through activation of different intracellular pathways, downstream of their binding with acetylated and/or oxidized LDL (Zani et al., 2015; Paquette et al., 2017b). It is now known that the concomitant presence of pathogenic variants in the oxidized-LDL receptor 1 (OLR1) gene in HeFH patients indicates higher CAD risk (Paquette et al., 2017b). Additionally, the recent discovery of a different metabolic pathway in endothelial cells, involving LDL uptake and transcytosis into endothelial cells through the acting-like kinase 1 (ALK1), in the absence of LDLR, has drawn more attention toward LDLR-independent mechanisms in order to more successfully address LDL accumulation and atherosclerosis (Kraehling et al., 2016).

Despite its relatively high prevalence and the well-established impact on CVD, FH is often underdiagnosed in clinical practice (deGoma et al., 2016; Knickelbine et al., 2016; Ershova et al., 2017). Moreover, although a relatively broad arsenal of therapeutic options is available, FH still remains a frequently under-treated condition (Benn et al., 2012; Lahtinen et al., 2015; Knickelbine et al., 2016; Ershova et al., 2017; Zamora et al., 2017). Taking into account the CVD-related burden on public health and the related health care expenditures (annual related costs that reach 192 billion Euro in the EU), it becomes evident that the effective management of FH poses a great challenge in clinical practice (European Association for Cardiovascular Prevention and Rehabilitation et al., 2011; Goldberg et al., 2011; Ito and Watts, 2015).

## FH genetics

The genetic diversity of FH results in significant phenotypic variability, rendering the diagnosis challenging, while it further highlights the need for individualized treatment strategies (Foody and Vishwanath, 2016). It is now clear that the underlying pathogenic mechanism in FH is the defective LDL clearance (Turgeon et al., 2016). The monogenic dominant inherited form is the most common type, comprising of three different pathogenic variants, namely of the LDLR gene, apolipoprotein B (ApoB), and pro-protein convertase subtilisin/kexin type 9 (PCSK9) (Hovingh et al., 2013; Turgeon et al., 2016). Furthermore, recent studies report pathogenic variants in the ApoE and Stap1 genes as extremely rare causes of autosomal dominant FH (Defesche et al., 2017; Pirillo et al., 2017). In addition to the dominant form of inheritance, FH may also present due to a very rare autosomal recessive form caused by a mutation in the LDLR adaptor protein 1 (LDLRAP1) gene (Garcia et al., 2001).

LDLR is the main cell membrane receptor for LDL in hepatocytes and its role is to remove LDL from the circulation through internalization of this ligand-receptor complex (Huff et al., 2014; Bouhairie and Goldberg, 2015; Turgeon et al., 2016). These receptors are recycled many times before specific enzymes (e.g., PCSK9 and IDOL) lead to their lysosomal degradation (Huff et al., 2014). To date, according to the British Heart Foundation (BHF) database, 1741 allelic variations of the LDLR have been registered, with 73.5% of them being substitutions (www.ucl.ac.uk/ldlr/LOVDv.1.1.0/). The classification (Class 1 through 5) of the different pathogenic variants includes: completely absent receptors, blocked transport to the Golgi apparatus, dysfunctional receptors or defective internalization, and recycling (Hartgers et al., 2015).

The primary screening target for FH is the LDLR pathogenic variants, accounting for more than 90% of the FH cases (Hartgers et al., 2015). In the remaining cases, the second in prevalence gene mutation involves ApoB (2–5% of cases) (Patel et al., 2015), an apolipoprotein that is found on each LDL particle and is responsible for the specific ligand-receptor binding and the subsequent clearance of LDL from the circulation (Walldius and Jungner, 2004). In these cases, the mutant apolipoprotein B-100 (specific for LDL, IDL, and VLDL) impairs the binding of the ApoB-containing particles by the LDLR in the liver, resulting in their accumulation in the systemic circulation which further triggers atherogenesis (Walldius and Jungner, 2004; Patel et al., 2015).

PCSK9 constitutes the third gene implicated in LDL metabolism in FH, mediating the LDLR degradation in lysosomes. Thus, PCSK9 pathogenic variants with either gain- or loss-of-function directly affect the LDL availability in the bloodstream. Indeed, gain-of-function pathogenic variants, which result in increased LDLR degradation, account for < 1% of the FH cases (Patel et al., 2015).

Finally, the aforementioned rare autosomal recessive type of FH is associated with the loss-of-function pathogenic variants of the LDLRAP1. This protein is involved in clathrin-dependent internalization/endocytosis of the LDLR, hence, these pathogenic variants also attenuate the LDL clearance from the circulation (Garcia et al., 2001; Rader et al., 2003).

## FH epidemiology

Current epidemiological data on the prevalence of HeFH vary according to the screened population (Goldstein et al., 1973; Mabuchi et al., 1977; Moorjani et al., 1989; Seftel et al., 1989; Slimane et al., 1993; Steyn et al., 1996; Vuorio et al., 1997; Kalina et al., 2001; Austin et al., 2004; Benn et al., 2012; Nordestgaard et al., 2013; de Ferranti et al., 2016; Pang et al., 2016; Safarova et al., 2016; Zhou and Zhao, 2016; Casula et al., 2017; Ershova et al., 2017; Zamora et al., 2017). As such, previous data have reported a HeFH prevalence of 1:500 in Caucasian MI survivors (Goldstein et al., 1973), while more recent studies showed rates of 1:137 in an unselected Danish population sample (Benn et al., 2012), and 1:192 in a Catalan database sample (Zamora et al., 2017). Similarly, the SEARCH Study reported a HeFH prevalence rate of 1:310 in a US population applying an e-phenotyping algorithm on electronic health care records (Safarova et al., 2016), whilst the 2016 US NHANES study reported a rate of 1:250 (de Ferranti et al., 2016). It should be noted that the different prevalence rates in various ethnic populations are also partly attributed to the lack of uniformity in the criteria used for FH diagnosis, the genotypic/phenotypic FH variations which might make the correct diagnosis challenging, as well as the different awareness and education/training worldwide (Goldberg et al., 2011; Benn et al., 2012; EAS Familial Hypercholesterolaemia Studies Collaboration et al., 2016; Foody and Vishwanath, 2016; Zhou and Zhao, 2016; Casula et al., 2017). Table 1 summarizes the available data on the reported HeFH prevalence rates in different countries/ethnic populations.

**Table 1:** Reported prevalence rates of heterozygous familial hypercholesterolemia (HeFH) and homozygous familial hypercholesterolemia (HoFH) in various countries/ethnic populations.

**Country/Ethnic population [References]**	**HeFH prevalence rate**
United States 1973 (Goldstein et al., 1973)	1:500
United States 2016, SEARCH Study (Safarova et al., 2016)	1:310
United States 2016, NHANES Study (de Ferranti et al., 2016)	1:250
Québécois French Canadians (Moorjani et al., 1989)	1:270
Tunisia (Slimane et al., 1993)	1:165
Finnish North Karelia (Vuorio et al., 1997)	1:441
Hungary (Kalina et al., 2001)	1:538
United Kingdom (Austin et al., 2004)	1:623
Denmark (Benn et al., 2012)	1:137
Catalan (Zamora et al., 2017)	1:192
Australia (Pang et al., 2016)	1:267
Japan (Mabuchi et al., 1977)	1:900
China (Zhou and Zhao, 2016)	1:212–1:357
Lebanon (Austin et al., 2004)	1:85
South Africa/Afrikaners (Steyn et al., 1996)	1:72
Ashkenazi Jews (Seftel et al., 1989)	1:67
West Siberian (Russian Federation) (Ershova et al., 2017)	1:108
**Country/Ethnic population [References]**	**HoFH prevalence rate**
Netherlands (Dutch) (Sjouke et al., 2015)	1:300,000
Catalan (Zamora et al., 2017)	1:425,774
Spain (Sanchez-Hernandez et al., 2016)	1:450,000
Québécois French Canadians (Moorjani et al., 1989)	1:275,000

Moreover, prevalence rates of 1:1,000,000 have been previously reported for HoFH (Nordestgaard et al., 2013). However, more recently, Sjouke et al. suggested a higher prevalence of approximately 1:300,000 based on a Dutch population sample (Sjouke et al., 2015), while a database analysis of Catalan patients reported a rate of 1:425,774 (Zamora et al., 2017). Similarly, another Spanish study estimated the HoFH prevalence at 1:450,000 (Sanchez-Hernandez et al., 2016), while the reported HoFH prevalence in Québécois French Canadians was 1:275,000 (Moorjani et al., 1989) (Table 1). Considering that < 1% of the FH population is detected in most countries worldwide and that CVD constitutes the first cause of death globally, such data on the FH prevalence appear not only reasonable, but further highlight the possibility that the actual undetected FH prevalence is even higher (Nordestgaard et al., 2013).

## Clinical findings in FH

The most common clinical findings in FH patients include tendon xanthomas, xanthelasmas (seen under the age of 25), and the corneal arcus (under the age of 45) (Bouhairie and Goldberg, 2015), with the former being considered specific and diagnostic for FH (European Association for Cardiovascular Prevention and Rehabilitation et al., 2011). However, not all FH patients present with clinical signs (Bouhairie and Goldberg, 2015).

These findings are considered related to the storage of circulating cholesterol inside macrophages of the extracellular matrix inside the tendons or the skin (Kim and Han, 2013; Soslowsky and Fryhofer, 2016). The Achilles tendon and the extensor tendons of the dorsum of the hands constitute the most common sites for tendon xanthomas; however, xanthomas may also involve the feet, elbows and antecubital fossae, knees, and buttocks (European Association for Cardiovascular Prevention and Rehabilitation et al., 2011; Kim and Han, 2013; Soslowsky and Fryhofer, 2016). Notably, tendon xanthomas can progress from thickening to tendon deposits, leading to significant changes in tendon biomechanics (Kim and Han, 2013; Soslowsky and Fryhofer, 2016).

It is also important to note that, although tendon xanthomas are specific/diagnostic for FH, lipid profile assessments should always be part of the diagnostic approach since there is also the rare possibility of diseases with either normal cholesterol (e.g., cases of cerebrotendinous xanthomatosis) (Parente et al., 2016), or normal/high cholesterol (e.g., sitosterolemia), which is responding extremely well to low cholesterol diet and bile acid sequestrants and it could be perhaps suspected in patients with poor response to statins, especially if these also fit the whole clinical picture (Yoo, 2016).

Xanthelasmas and arcus cornealis are the two features/signs that may be noted from the examination of the ocular area and eyes in FH patients. The former represents deposition of cholesterol around the eyelids, usually near the inner canthus. Further examination of the patient's cornea may reveal also a brighter zone around the rim, i.e., the arcus cornealis (Kim and Han, 2013). These findings further reflect the degree of the underlying atherosclerotic damage throughout the vasculature, and thus, their presence on clinical examination should alert clinicians and prompt the early diagnosis and treatment of FH (Hovingh et al., 2013; Hartgers et al., 2015; Ito and Watts, 2015).

## FH diagnosis: diagnostic criteria and approach

FH still remains greatly underdiagnosed (Hovingh et al., 2013), despite the fact that there are several diagnostic criteria/systems which can be reliably applied in everyday clinical practice once an alarming family/personal history or suspicious clinical sign(s) are noted. Such systems must be applied promptly in order to lead to early diagnosis and treatment of FH patients, preventing disease progression and ASCVD. Hence, increased awareness is needed among clinicians, especially in primary care, in order to tackle this problem in routine practice.

Notably, the existing diagnostic systems (mostly scoring algorithms) for FH entail slightly different criteria which vary on the proposed biochemical values/cut offs, but their prediction value is relatively similar (European Association for Cardiovascular Prevention and Rehabilitation et al., 2011; Hartgers et al., 2015). According to the European 2016 guidelines, FH should be suspected when patients present with premature CVD (men < 55 y.o. and women < 60 y.o.), have a family history of premature CVD, have a family history of tendon xanthomas, and when their LDL is >5 mmol/l (190 mg/dl), or LDL >4 mmol/l (150 mg/dl) in children (Catapano et al., 2016).

Currently, the main diagnostic systems for FH include: the US Make Early Diagnosis to Prevent Early Death (MEDPED) and WHO criteria (Table 2); the UK Simon Broome system (UK FH Register criteria) (Table 3); the Dutch Lipid Network Criteria (Table 4); the National Lipid Association (NLA) expert panel recommendations (Table 5); as well as the Japanese FH diagnostic criteria (Table 6) (European Association for Cardiovascular Prevention and Rehabilitation et al., 2011; Harada-Shiba et al., 2012b; Hartgers et al., 2015; Turgeon et al., 2016). Of these, the Simon Broome criteria indicate a possible or definite diagnosis, while the Dutch Lipid Network criteria, as well as the US MEDPED and WHO system calculate a diagnostic score (Health Quality Ontario, 2007; European Association for Cardiovascular Prevention and Rehabilitation et al., 2011).

**Table 2:** MEDPED and WHO criteria for FH diagnosis.

**US MEDPED and WHO CRITERIA for FH diagnosis**	**SCORE**
**FAMILY HISTORY**	
First degree relative with premature CAD and/or LDL >95th centile	1
First degree relative with tendon xanthomas and/or children < 18 with LDL >95th centile	2
**CLINICAL HISTORY**	
Premature CAD	2
Premature cerebral/peripheral vascular disease	1
**PHYSICAL EXAMINATION**	
Tendon xanthomas	6
Arcus cornealis < 45 y.o	4
**LDL**	
>8.5 mmol/l (>330 mg/dl)	8
6.5–8.4 mmol/l (250–329 mg/dl)	5
5–6.4 mmol/l (190–249 mg/dl)	3
4–4.9 mmol/l (155–189 mg/dl)	1
**DIAGNOSIS ACCORDING TO OVERALL SCORE**	
Definite	>8
Probable	6–8
Possible	3–5
Unlikely	< 3

*FH, Familial hypercholesterolemia; MEDPED, Make early diagnosis to prevent early death; WHO, World health organization; LDL, Low density lipoprotein; CAD, Coronary artery disease*.

**Table 3:** Simon Broome criteria for diagnosis of familial hypercholesterolemia.

**Simon Broome criteria for FH diagnosis**	
1. In adults: TC >7.5 mmol/L (or, when available, LDL >4.9 mmol/L) and in pediatric patients: TC >6.7 mmol/L, or LDL >4.0 mmol/L, and	DEFINITE
2. Tendon xanthoma in the patient or first/second degree relative, or alternatively:	
3. Presence of LDL-receptor, ApoB, or PCSK9 mutation	
1. In adults: TC >7.5 mmol/L (or, when available, LDL >4.9 mmol/L) and in pediatric patients: TC >6.7 mmol/L, or LDL >4.0 mmol/L, and	POSSIBLE
2. Family history of MI < 50 y.o. in second degree relative or < 60 y.o. in first degree relative or, alternatively,	
3. Family history of TC >7.5 mmol/L in a first- or second-degree relative	

*FH, Familial hypercholesterolemia; TC, Total cholesterol; LDL, Low density lipoprotein; MI, Myocardial infarction; ApoB, Apolipoprotein B; PCSK9, Proprotein convertase subtilisin/kexin type 9; y.o., Years old*.

**Table 4:** Dutch Lipid Network criteria for diagnosis of familial hypercholesterolemia.

**DUTCH LIPID NETWORK**	**SCORE**
**FAMILY HISTORY**	
Premature CVD (men < 55 y.o., women < 60 y.o.) in first degree relative, or	1
LDL >95th percentile in first degree relative and/or	1
Tendon xanthoma and/or arcus cornealis in first degree relative, or	2
LDL >95th percentile in children < 18 y.o.	2
**PERSONAL HISTORY**	
Premature CAD in patient (men < 55 y.o., women < 60 y.o.), or	2
Premature cerebral or peripheral vascular disease (men < 55 y.o., women < 60 y.o.)	1
**CLINICAL EXAMINATION**	
Tendon xanthomas, or	6
Arcus cornealis < 45 y.o.	4
**LDL**	
≥8.5 mmol/l (≥330 mg/dl)	8
6.5–8.4 mmol/l (250–329 mg/dl)	5
5–6.4 mmol/l (190–249 mg/dl)	3
4–4.9 mmol/l (155–189 mg/dl)	1
Presence of functional LDLR mutation (in the LDLR, ApoB or PCSK9 gene)	8
**DIAGNOSIS ACCORDING TO OVERALL SCORE**	
Definite	>8
Probable	6–8
Possible	3–5
Unlikely	< 3

*FH, Familial hypercholesterolemia; CVD, Cardiovascular disease; CAD, Coronary artery disease; LDL, Low density lipoprotein; LDLR, Low density lipoprotein receptor; ApoB, Apolipoprotein B; PCSK9, Proprotein convertase subtilisin/kexin type 9; y.o., Years old*.

**Table 5:** National Lipid Association (NLA) diagnostic criteria for familial hypercholesterolemia.

**NLA diagnostic criteria for FH**	
**Children, adolescents, young adults <20 y.o**.	**Adults ≥20 y.o**.
LDL ≥4.1 mmol/l (160 mg/dl)	LDL ≥4.9 mmol/l (190 mg/dl)
Non-HDL ≥4.9 mmol/l (190 mg/dl)	Non-HDL ≥5.7 mmol/l (220 mg/dl)

*FH, Familial hypercholesterolemia; NLA, National lipid association; LDL, Low density lipoprotein; HDL, High density lipoprotein*.

**Table 6:** Japanese diagnostic criteria for familial hypercholesterolemia.

**Japanese diagnostic criteria for FH**
1. Pre-treatment LDL ≥180 mg/dl (≥4.6 mmol/l)
2. Tendon xanthoma, or nodular skin xanthoma
3. Family history (within the second degree relatives): FH or premature CAD

*FH, Familial hypercholesterolemia; LDL, Low density lipoprotein; CAD, Coronary artery disease*.

The NLA criteria may be useful for FH detection in childhood, and clinicians should keep in mind that children with LDL levels ≥4.1 mmol/l (≥160 mg/dl) are most probably diagnosed with FH (Ito and Watts, 2015). In order to avoid false negative results due to high HDL obscuring LDL levels in HeFH, screening should start after the first 6 weeks of life. Notably, high LDL levels are expected throughout childhood and adulthood, with the exception of pubertal years, when the growth spurt takes place (European Association for Cardiovascular Prevention and Rehabilitation et al., 2011).

Another important parameter in the diagnosis of FH is the family history. As the index patient may present for investigations before any other family member(s) develop CVD or with unknown/unclear family CVD history, this factor can be frequently underestimated. This also constitutes a problem when dealing with populations/patient groups with already high CVD prevalence (European Association for Cardiovascular Prevention and Rehabilitation et al., 2011).

Finally, FH diagnosis could be confirmed by genetic testing (European Association for Cardiovascular Prevention and Rehabilitation et al., 2011). Investigation of the monogenic form of FH includes testing for pathogenic variants in the genes for LDLR, ApoB, PCSK9 (related with the autosomal dominant type), and LDLRAP1 (autosomal recessive form) (Harada-Shiba et al., 2012a; Ito and Watts, 2015). Till recently, with the different detection techniques used, 30% of patients with a definite FH diagnosis were suspected to be missed due to the high variability of the underlying pathogenic variants (Watts et al., 2015). Next generation sequencing (NGS) seems a promising technique as far as detection rates are concerned (Bell and Watts, 2016) and it is now the method of choice for FH detection in the UK, recommended by NICE, as it is proven to be cost-effective (https://www.nice.org.uk/guidance/cg71/evidence/surveillance-review-decision-june-2015-pdf-2361738349).

Through simultaneous screening of multiple genes this technique can identify known and novel causative pathogenic variants for FH, helping scientists to know more about FH (Hartgers et al., 2015; Watts et al., 2015). This would be particularly useful for understudied populations and should be performed by appropriate laboratories in order to classify the findings as benign/pathogenic/of unknown significance (Hartgers et al., 2015; Watts et al., 2015) and subsequently investigate the significance of the rare or unknown variants if present (Reiman et al., 2016). Of note, in cases where no pathogenic variant is detected in the four genes (LDLR, ApoB, PCSK9, and LDLRAP1), NGS is expected to successfully differentiate the polygenic type of the disease through whole/targeted genome, or whole exome sequencing (Hartgers et al., 2015; Bell and Watts, 2016), which is performed through the Genomics England 100K Genomes Project since 2013 in the UK (Turnbull et al., 2018). Braenne et al. confirmed the significance of exome sequencing in detecting small nucleotide variants and large rearrangements leading to FH phenotypes in 2016, and highlighted the need of co-segregation analysis in order to identify the role of these variants. As the FH diagnosis is often missed, even in CAD patients, it is suggested that systematic and organized variant analysis is applied in the future (Brænne et al., 2016).

As aforementioned, according to the UK NICE guidelines, genetic testing could guide clinicians toward accurate diagnosis and prognosis, as well as timely management in FH cases. However, although genetic testing is considered to be cost-effective, it should not be overlooked that the circulating LDL levels determine the associated CVD risk and not the mutation itself (Hovingh et al., 2013). Of note, HDL levels may be also found normal or low, potentially due to increased ApoA-I turnover and catabolism of dysfunctional HDL by the ApoE-receptor (Ooi et al., 2013). Moreover, due to the high number of pathogenic variants and the low detection rates of the existing methods, genetic testing for FH remains inadequate in countries with greater genetic heterogeneity (Health Quality Ontario, 2007; Haralambos et al., 2016; Sharifi et al., 2016; Fairoozy et al., 2017).

It is worth noting that reaching the diagnosis may be particularly complicated in FH cases due to the polygenic inherited form. It is now known that many hypercholesterolemia cases with none of the above pathogenic variants are related to small-effect LDL-raising alleles (Talmud et al., 2013). The development of a 12-single nucleotide polymorphism (12-SNP) score has been found successful in differentiating healthy individuals from FH patients without one of the three common pathogenic variants (LDLR, ApoB, and PCSK9; Futema et al., 2015). The polygenic type is also characterized by lower LDL levels due to its more benign nature, which can be deceiving and lead to false negative results (Hartgers et al., 2015).

When genetic testing is not available or when a common pathogenic variant is not found, the diagnosis should be based on the LDL levels, the presence of atherosclerotic disease, response to treatment, and family history (Ito and Watts, 2015; Watts et al., 2015).

**Figure 1:**
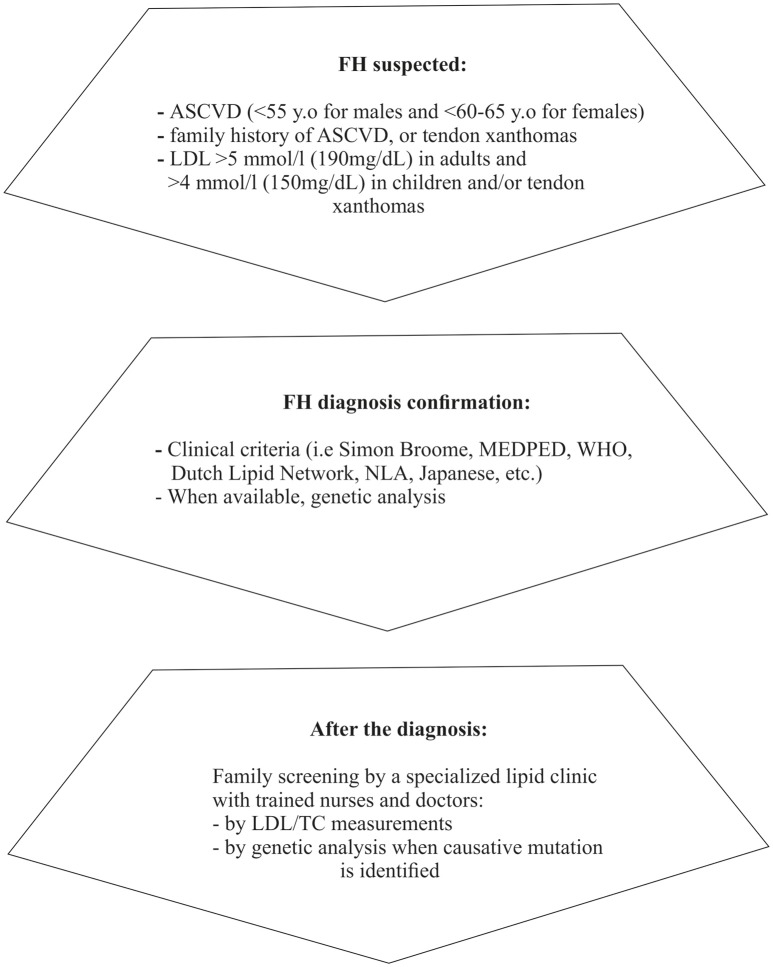
Diagnostic approach/steps for suspected FH (based on Catapano et al., 2016; Jellinger et al., 2017). ASCVD, Atherosclerotic cardiovascular disease; LDL, Low density lipoprotein; MEDPED, Make early diagnosis to prevent early death; WHO, World health organization; NLA, National lipid association; LDL, Low density lipoprotein; TC, Total cholesterol.

Finally, fasting lipid profile for FH testing should preferably be avoided during acute illness (Watts et al., 2015) or chronic concomitant illnesses (e.g., hypothyroidism, diabetes, liver, and renal impairment) and certain medications should be excluded as potential secondary causes (Hovingh et al., 2013; Hartgers et al., 2015). A concise diagnostic algorithm is summarized in Figure 1.

## CVD risk assessment in FH

Once FH is diagnosed, a comprehensive CVD risk assessment should be performed, since the prevalence of CAD among FH patients can reach 33% (Benn et al., 2012). It is now clearly demonstrated that carriers of FH pathogenic variants present increased CVD risk when compared to non-carriers, regardless of the LDL level, due to their persistent lifelong hypercholesterolemia (Khera et al., 2016). It should be stressed that, although the existing CVD risk assessment tools are helpful in the general population, these are usually not able to accurately predict the long-term CVD risk in FH patients. As such, the arterial damage from the chronic exposure to high cholesterol levels is generally under-estimated (Bouhairie and Goldberg, 2015). Interestingly though, a recent cross-sectional cohort study demonstrated that the inclusion of age, HDL, gender, hypertension, and smoking in the context of a cumulative clinical score, named as the Montreal-FH-SCORE, can predict the CVD risk in FH patients, regardless of their LDL levels (Paquette et al., 2017a). It is also noteworthy that, Apo A-I and ApoB apolipoproteins are considered as better CVD risk predictors than LDL, even in patients who are on lipid-lowering treatment (Walldius and Jungner, 2004).

Of note, triglycerides (TG) are not included in the diagnostic criteria for FH, as their abnormal metabolism is not directly linked to the disease. However, they are recognized as an independent CVD risk factor and they are part of the general management goals for CVD prevention (Catapano et al., 2016; Jellinger et al., 2017). Thus, it is recommended that FH patients would benefit from intensive advice against all risk factors, including TG (Catapano et al., 2016).

Moreover, screening should evaluate the overall CVD risk based on the smoking status, body mass index (BMI) and lipid, glucose, and blood pressure profile of the patient, whilst taking into account the presence of any atherosclerotic disease (Watts et al., 2015). In HeFH, these variables predict the age of onset and extent of CVD (Neil et al., 2008). Moreover, it has been shown that the total cholesterol (TC) burden, calculated as TC multiplied by age at diagnosis, plus annually assessed TC is linked directly to coronary calcification (Gallo et al., 2017).

Arterial imaging (echocardiogram, cardiac computed tomography, and angiography) can be used in order to evaluate more accurately the degree of subclinical CVD (Walus-Miarka et al., 2016). Such assessments should be offered at least to high risk patients, since 25–90% of asymptomatic FH patients have atherosclerotic plaques on carotid ultrasound (Khan et al., 2011; van den Oord et al., 2013). Furthermore, a study in 40 FH patients has also showed that their Carotid Intima-Medial Thickness (CIMT) is significantly higher (0.7–1.83 mm) compared to controls (0.48–0.73 mm) (Khan et al., 2011). However, the significance of monitoring CIMT is not established yet, since CIMT regression does not appear to directly correlate with CVD outcomes, and coronary artery calcium might be of greater prognostic value (Costanzo et al., 2010; Phan et al., 2014).

It is also worth noting that in pediatric FH cases the presence of diabetes or Kawasaki disease with large aneurysms or chronic kidney disease confers significantly higher risk and, thus, these patients need even more intensified treatment (Watts et al., 2015).

Recent guidelines recommend including lipoprotein (a) [Lp(a)] in the CVD risk assessment of all FH patients (Watts et al., 2015; Catapano et al., 2016), since Lp(a) levels 3.5-fold higher than normal induce atherogenesis and increase the CVD risk, especially in those with high LDL levels (Bucci et al., 2016). Lp(a) is more abundant in HoFH, but it is also high in HeFH (Sjouke et al., 2017). Existing data indicate that the LDLR impairment is not responsible for the atherogenic profile of Lp(a), but the underlying mechanisms are still not completely understood (Cuchel et al., 2014b). Interestingly, Lp(a) has both independent and synergistic effects to LDL on the cumulative CVD risk [National Cholesterol Education Program (NCEP) Expert Panel on Detection, Evaluation, and Treatment of High Blood Cholesterol in Adults (Adult Treatment Panel III), 2002; Jacobson, 2013]. Being an independent CVD risk factor, this parameter should be taken into account when setting the LDL targets for each FH patient [National Cholesterol Education Program (NCEP) Expert Panel on Detection, Evaluation, and Treatment of High Blood Cholesterol in Adults (Adult Treatment Panel III), 2002]. Notably, as will be described in the following sections, niacin has been shown to effectively reduce Lp(a) (Hovingh et al., 2013), but its use is limited due to its side effects (Bucci et al., 2016), whilst other Lp(a)-lowering medications include PCSK9 inhibitors, ApoB antisense oligonucleotides, selective second generation apo(a) antisense oligonucleotides, CETP inhibitors, thyroid hormone receptor agonists, estrogens, and IL-6R mAbs (Ellis et al., 2017). Currently, the treatment of choice for very high Lp(a) levels is LDL apheresis (Bucci et al., 2016).

## Organizing cascade screening in FH

After confirming the FH diagnosis of the screened patient (“index” case), cascade screening of the patient's family should be arranged by a trained health care professional in order to identify any relatives with the disease (Watts et al., 2015; Bell and Watts, 2016). Screening involves assessing personal history for the presence of hypercholesterolemia, phenotypic traits, and premature CVD, along with a fasting lipid profile (Watts et al., 2015). Strong collaboration between lipid specialist clinics and general practitioners is essential for this system to work effectively (Watts et al., 2015; Bell and Watts, 2016). Genetic testing is not currently considered mandatory (Ito and Watts, 2015); however, the current UK NICE guidelines suggest that it is cost-effective (Kerr et al., 2017). When the mutation is known, it could be used to continue the cascade screening (Catapano et al., 2016). Of note, when a relative of the index case is diagnosed with hypercholesterolemia/positive mutation, their own first-degree relatives should be subsequently screened (Hovingh et al., 2013; Catapano et al., 2016). Approximately half of the screened relatives are expected to have FH due to the autosomal dominant way of inheritance in most cases (European Association for Cardiovascular Prevention and Rehabilitation et al., 2011; Bell and Watts, 2016).

In pediatric FH cases, according to the International FH Foundation, it is generally recommended that screening should start before the age of 10, usually between the age of 5 and 10 (Watts et al., 2015). The American College of Endocrinology suggests consecutive screening of children at the ages of 3, 9, 11, and 18 (Jellinger et al., 2017), and for adolescents >16 y.o. every 5 years or even more frequently if high CVD risk is suspected (Jellinger et al., 2017). Diagnosis of HeFH is considered most likely even in the absence of positive family history in children >2 y.o. with LDL ≥5 mmol/l (193 mg/dl), while HoFH is usually the case in children < 10 y.o. with LDL >13 mmol/l (Watts et al., 2015). In cases of hypercholesterolemia or premature CVD family history the diagnosis is set at LDL >4 mmol/l (155 mg/dl) and at LDL ≥3.5 mmol/l (135 mg/dl) when a parent has been diagnosed genetically (Catapano et al., 2016).

## Management of FH

FH patients should be advised that life-long management with regular follow up will be required, since at the moment there are no curative treatment options. In clinical practice, treatment must be initiated as soon as possible, aiming to lower lipidemia, particularly LDL (Turgeon et al., 2016; Migliara et al., 2017), at levels which reduce the overall CVD risk to that of the general population (Goldberg et al., 2011). As such, an individualized management plan is required, taking into consideration that typically the overall treatment approach for HoFH should be more aggressive compared to HeFH (European Association for Cardiovascular Prevention and Rehabilitation et al., 2011; Bouhairie and Goldberg, 2015; Watts et al., 2015).

### Setting the targets for FH treatment

Current strategies in the management of FH focus on specific LDL targets, since compiling clinical evidence shows improved outcomes through this approach (European Association for Cardiovascular Prevention and Rehabilitation et al., 2011). Meta-analysis data from 26 randomized controlled trials (RCTs) showed that reduction of LDL by 1 mmol/l (40 mg/dl) directly correlates with a 22% CVD risk reduction (Cholesterol Treatment Trialists et al., 2010). Accordingly, although different treatment guidelines exist globally, a common treatment goal in clinical practice is to achieve a 50% reduction of the initial LDL levels (European Association for Cardiovascular Prevention and Rehabilitation et al., 2011; Goldberg et al., 2011; Hovingh et al., 2013; Bouhairie and Goldberg, 2015; Hartgers et al., 2015). Of note, LDL targets should be the same for both HeFH and HoFH patients (Najam and Ray, 2015), although clinicians should keep in mind that FH patients at higher overall CVD risk would benefit from more aggressive treatment (Table 7). According to the 2016 European guidelines for FH management, the recommended LDL target is < 2.6 mmol/l (100 mg/dl) or < 1.8 mmol/l (70 mg/dl) in cases with concomitant CVD (Catapano et al., 2016) (Table 8).

**Table 7:** Candidates for familial hypercholesterolemia treatment intensification.

**Candidates for FH treatment intensification**
(1) Patients with established arterial disease
(2) Patients with diabetes
(3) Patients with family history of premature CVD (< 45 y.o. males and/or < 55 y.o. females),
(4) Smokers
(5) Patients with ≥2 risk factors for CAD
(6) Patients with Lp(a) >50 mg/dl
(7) Patients with LDL >4.1 mmol/l (>160 mg/dl) and non-HDL >4.9 mmol/l (>190 mg/dl)
(8) Patients not able to achieve the target of 50% LDL reduction

*FH, Familial hypercholesterolemia; CVD, Cardiovascular disease; CAD, Coronary artery disease; LDL, Low density lipoprotein; HDL, High density lipoprotein; Lp(a), lipoprotein (a). Adapted from Bouhairie and Goldberg (2015) and Goldberg et al. (2011)*.

**Table 8:** Therapeutic targets for familial hypercholesterolemia (2016 ESC/EAS Guidelines for the Management of Dyslipidemias).

**Therapeutic targets for FH**	
**Patient population**	**LDL target**
Children	<3.5 mmol/l (<135 mg/dl)
Adults without established CVD	<2.6 mmol/l (<100 mg/dl)
Adults with established CVD	<1.8 mmol/l (<70 mg/dl)

*FH, Familial hypercholesterolemia; CVD, Cardiovascular disease; EAS, European atherosclerosis society; ESC, European Society of Cardiology; LDL, Low density lipoprotein; Adapted from Catapano et al. (2016)*.

Despite such guidelines and the well-established benefits of the proposed target LDL levels, existing evidence indicates that often FH patients remain under-treated. Indeed, a cross-sectional study from the Netherlands on the management of HeFH in outpatient clinics revealed that most of these patients failed to achieve the desirable 2.5 mmol/l threshold for LDL, since treating physicians were accepting higher LDL levels with less intensified treatment (Pijlman et al., 2010).

However, it is also not uncommon for FH patients with very high pre-treatment LDL levels to fail reaching the desired treatment targets despite intensified therapy. In such cases, new realistic targets should be set with regular follow up, and, as a general rule of practice, clinicians should aim for the maximum LDL reduction with minimum side effects (European Association for Cardiovascular Prevention and Rehabilitation et al., 2011; Hovingh et al., 2013).

Finally, HDL has been identified as an additional parameter which should be taken into account when planning the treatment approach for FH patients. Currently, specific HDL targets are not included in the existing clinical practice recommendations. However, due to its beneficial role in reverse cholesterol transport and its antioxidant/cardio-protective effects, higher HDL levels are desirable (HDL ≥60 mg/dl), and this has been shown to enhance the benefits of lowering LDL [National Cholesterol Education Program (NCEP) Expert Panel on Detection, Evaluation, and Treatment of High Blood Cholesterol in Adults (Adult Treatment Panel III), 2002].

### Lifestyle changes and a holistic approach in the management of FH

A holistic approach with a spectrum of lifestyle changes should be adopted in the long-term management plan for FH patients, aiming to optimize LDL levels (Goldberg et al., 2011) and reduce the overall CVD risk (Arsenault et al., 2017).

#### Weight control and FH

Taking into account the detrimental effects of increased abdominal adiposity on the production of small dense LDL, HDL, and on other parameters (e.g., pro-inflammatory circulating adipokines) which collectively increase the CVD risk (Tchernof and Despres, 2013), FH patients should be advised to maintain a BMI within the normal range (European Association for Cardiovascular Prevention and Rehabilitation et al., 2011; Goldberg et al., 2011; Watts et al., 2015). For obese FH patients, an individualized strategy should be tailored to achieve and maintain the desired weight loss through a multidisciplinary approach (e.g., specialized input from dieticians and advice for appropriate exercise; Goldberg et al., 2011; Najam and Ray, 2015).

#### Dietary interventions in FH

Referral of FH patients for specialized dietary/nutritionist advice is helpful in their long-term management (Najam and Ray, 2015). A diet low in saturated fats (total daily fat intake in the range of 25–35%, with saturated fats < 7% of overall intake), low in cholesterol (< 200 mg/day) and high in fiber (10–20 g/day) is generally advised for FH patients (Goldberg et al., 2011; Hovingh et al., 2013; Cuchel et al., 2014b). Of note, such dietary approaches/counseling in children with FH have also been associated with improved lipid patterns (Torvik et al., 2016). Of note, dietary fibers are known to lower LDL and TC (Brown et al., 1999; Hartley et al., 2016), as well as diastolic pressure (Hartley et al., 2016), potentially acting through altering cholesterol absorption and specific hormonal signaling (Van Horn, 1997); however, conclusive data on their long-term CVD impact/outcomes are still insufficient (Malhotra et al., 2014; Hartley et al., 2016).

Overall, following a diet low in saturated fat has been shown to decrease LDL levels by 8–10%, while limiting cholesterol consumption to < 200 mg daily appears to lead to a 3–5% LDL reduction (Hovingh et al., 2013). However, diet alone is not sufficient to significantly alter the progression of the disease in most FH cases (Cuchel et al., 2014b).

Regarding specific dietary patterns, existing data suggest that adhering to the Mediterranean diet may have multiple benefits. Indeed, the Mediterranean diet (low in saturated fat and high in monounsaturated fat and complex carbohydrates) has been associated with a 30% reduction in major CVD events compared to a low-fat diet alone (Barry et al., 2016).

Previous recommendations have also suggested daily consumption of plant sterols for cholesterol reduction (Malhotra et al., 2014). Plant sterols remove cholesterol from bile salt micelles and affect cholesterol absorption, hence they can play a role in cholesterol reduction (Hovingh et al., 2013). However, larger randomized RCTs are needed in order to assess their exact role in FH. A recent systematic review by Malhotra et al. that included 15 RCTs in FH patients compared the effect of cholesterol-lowering diets vs. other dietary interventions (e.g., plant sterols) on ischemic heart disease, as well as on the number of deaths and age at death (Malhotra et al., 2014). Overall, this systematic review reported no clear effect of these dietary interventions on the studied primary outcomes, with insufficient evidence to routinely recommend any of these in the management of FH (Malhotra et al., 2014).

#### Exercise and FH

Increased physical activity is generally advised as part of the overall management of dyslipidemias as it is associated with favorable impact on metabolic risk factors [National Cholesterol Education Program (NCEP) Expert Panel on Detection, Evaluation, and Treatment of High Blood Cholesterol in Adults (Adult Treatment Panel III), 2002; Jellinger et al., 2017]. Indeed, it seems to have a protective effect against atherosclerosis and oxidative stress, specifically preventing early endothelial dysfunction in LDLR-deficient mice (Guizoni et al., 2016). However, caution is necessary in FH patients with established arterial stenosis (ostial or aortic), due to the risk of impairing the underlying hemodynamic status (Cuchel et al., 2014b). In such cases, low-intensity exercise could be potentially recommended when considered safe, as even this can impact on the overall survival (Barry et al., 2016). For individuals who are safe to exercise, it is recommended that they adhere to 60 min of daily aerobic exercise plus muscle-strengthening twice a week (Jellinger et al., 2017).

#### Alcohol and FH

In the context of the long-term management and CVD risk reduction in FH, alcohol consumption should be limited (Goldberg et al., 2011). Light/moderate alcohol intake (up to 10 g daily) has been associated with lower CVD risk, mostly due to elevation of HDL and improved insulin sensitivity; however, high alcohol intake leads to increased CVD risk (de Jesus et al., 2016). According to the UK NICE guidelines, as for the general population, the recommended alcohol intake should not exceed 3–4 units/day for adult men and 2–3 units for women, while it is also important to avoid binge drinking (www.nice.org.uk/guidance/CG71).

#### Smoking cessation

Smoking represents another key factor in the plan for CVD risk reduction in FH patients (Goldberg et al., 2011), as it is known to affect several proteins which participate in the atherosclerosis process (Huang et al., 2016). This should be addressed rather aggressively in all FH patients who report smoking (Goldberg et al., 2011; Najam and Ray, 2015; Turgeon et al., 2016), and referral to specialized centers for smoking cessation should be offered, if necessary (Nordestgaard et al., 2013). Moreover, young patients with FH and their families should be thoroughly informed and strongly advised to avoid starting smoking (Nordestgaard et al., 2013).

#### Optimization of blood pressure

Optimizing the blood pressure control in FH patients should also not be overlooked as part of the approach to prevent premature atherosclerotic disease (Turgeon et al., 2016), since elevated blood pressure is a well-established CVD risk factor (Goswami and Manohar, 2016; Rust and Ekmekcioglu, 2016). Blood pressure targets for FH patients should be set at < 140/90 mmHg or at < 130/80 mmHg for patients with diabetes (Goldberg et al., 2011).

### Statin treatment in FH

Despite careful planning and strict adherence to a healthy lifestyle, the vast majority of FH patients will eventually require lipid-lowering drug therapy. Thus, drug therapy should be promptly initiated when after a trial period of lifestyle changes the levels of LDL and TC remain ≥4.9 mmol/l (≥190 mg/dl) and ≥5.7 mmol/l (≥220 mg/dl), respectively (Goldberg et al., 2011). Currently, statins represent the first step in the pharmacologic treatment of FH. Statin treatment should be ideally initiated at the age of 8–10 for children with HeFH, and as soon as the diagnosis is made for HoFH patients (Kolansky et al., 2008; Bouhairie and Goldberg, 2015).

Statins are selective HMG-CoA reductase inhibitors, resulting in LDL lowering (Hartgers et al., 2015; Watts et al., 2015). This is followed by the activation of the sterol regulatory element binding protein-2 (SREBP2), a transcription factor which subsequently up-regulates the expression of LDLR in hepatocytes (Huff et al., 2014). This up-regulation leads to enhanced clearance of LDL and other Apo-B containing lipoproteins from the circulation. Statins have been extensively studied in large clinical trials and have well-established benefits on CVD morbidity and mortality (European Association for Cardiovascular Prevention and Rehabilitation et al., 2011). Moreover, statins are also useful in secondary prevention, improving CVD outcomes in patients with established CAD (Hovingh et al., 2013).

Early intervention in HeFH with statins has shown to markedly decrease LDL levels (up to 60%), but this reduction can reach only up to 20% in HoFH patients (Hovingh et al., 2013). Of note, statin-treated FH patients have been shown to exhibit similar arterial imaging as subjects in the general population (Bos et al., 2017).

Furthermore, statins seem to increase HDL levels through blocking by 30% the activity of the cholesteryl ester transfer protein (CETP; a protein mediating the transfer of lipids between HDL and ApoB-containing particles; Postmus et al., 2016). The overall effect of statins on HDL levels appears to depend on genetic variations of the CETP locus (Postmus et al., 2016), as well as on the baseline HDL levels and the level of the HDL-bound anti-oxidative enzyme paraoxonase-1 (PON1) (Himbergen et al., 2005).

Among the available statins, pravastatin is approved for use in patients over 8 years old by the US Food and Drug Administration (FDA), whereas lovastatin, atorvastatin, simvastatin, and rosuvastatin can be used after the age of 10 (Bouhairie and Goldberg, 2015). The latter three statins together with pitavastatin are classified as moderate to high potency statins, and represent the first line choices in FH. Prescribing maximum doses of atorvastatin and rosuvastatin seems to be the general rule (European Association for Cardiovascular Prevention and Rehabilitation et al., 2011; Bouhairie and Goldberg, 2015).

It is noteworthy that, the pharmacokinetic properties of different statins vary significantly (Benes et al., 2016). Most of the statins are mainly metabolized in the liver by cytochrome P450 enzymes, with the exception of pravastatin, rosuvastatin, fluvastatin, and pitavastatin [National Cholesterol Education Program (NCEP) Expert Panel on Detection, Evaluation, and Treatment of High Blood Cholesterol in Adults (Adult Treatment Panel III), 2002; Benes et al., 2016], which should be preferred with concomitant use of CYP3A4 inhibitors (Benes et al., 2016). The lipid-lowering effect depends also on other factors, including the absorption, metabolism, dietary habits, compliance, genetic background, ApoE phenotype, gender and hormonal status [National Cholesterol Education Program (NCEP) Expert Panel on Detection, Evaluation, and Treatment of High Blood Cholesterol in Adults (Adult Treatment Panel III), 2002]. Accordingly, the effects of statin treatment on CIMT have been shown to differ based on the potency and dose of the prescribed statin, with aggressive treatment resulting in CIMT regression (Smilde et al., 2001).

In the context of an individualized management plan, it is suggested that FH patients are involved in the decision making process regarding statin treatment. As such, the overall CVD risk should be assessed, LDL-lowering targets should be agreed with the patient, and a statin that could potentially provide the desirable effect should be then initiated (European Association for Cardiovascular Prevention and Rehabilitation et al., 2011). Subsequent fine-tuning will be required until the LDL target is reached, with data suggesting that doubling of the statin dose can lead to a further reduction in LDL by 6% [National Cholesterol Education Program (NCEP) Expert Panel on Detection, Evaluation, and Treatment of High Blood Cholesterol in Adults (Adult Treatment Panel III), 2002].

Despite the established benefits of statin treatment in FH, current data indicate that statins are not offered to all FH patients in routine clinical practice, with a study in an unselected Danish population reporting that at least half of the FH patients were not on lipid-lowering treatment (Benn et al., 2012). Therefore, targeted efforts are still required in order to address various barriers to prompt and appropriate FH treatment.

Side effects from statin treatment constitute one of the key barriers/problems in the management of FH patients. Clinical trials involving medium-term follow-up of statins offered to children have concluded that these are both effective and safe (Goldberg et al., 2011). However, side effects such as myopathy (rarely rhabdomyolysis) and elevated liver enzymes (rarely hepatotoxicity) have been reported, without outweighing the overall significant benefit on CVD (Bouhairie and Goldberg, 2015). Notably, data from RCTs comparing statins against placebo on more than 129,000 patients show significant correlation of statin-induced side effects with advanced age, small body size, female gender, renal and liver impairment, hypothyroidism, perioperative time-frame, multi-organ pathology, and alcoholism (European Association for Cardiovascular Prevention and Rehabilitation et al., 2011).

Myopathy constitutes the most frequent (5–15%) side effect of statins, with rhabdomyolysis being its most dangerous form (Pasternak et al., 2002; Catapano et al., 2016). Muscle cell damage and death (rhabdomyolysis) result in the release of creatine phosphokinase (CK) and myoglobin among other intracellular molecules, while the accumulation of myoglobin in the kidneys can lead to renal failure and death (Pasternak et al., 2002; Tomaszewski et al., 2011; Catapano et al., 2016). Death associated with statin-induced rhabdomyolysis is considered extremely rare: < 1 death/million statin prescriptions (Pasternak et al., 2002) and 7.6% in patients with statin-related rhabdomyolysis (Holbrook et al., 2011), while case reports link these fatal events with concomitant medications, like cancer treatment (Nelson et al., 2017) and non-steroid anti-inflammatory drugs (Noordally et al., 2012). CK is commonly used as a marker to monitor muscle cell damage in statin-treated FH patients (Catapano et al., 2016).

Statin-induced myopathy could be justified genetically in certain cases, based on previous findings from the SEARCH genome-wide association study which has identified a SNP in the *SLCO1B1* gene as a potential risk factor (Stewart, 2013). Other SNPs associated with myopathy are the polymorphisms in the *ABCB1* and *ABCG2* genes (Ferrari et al., 2014).

In cases with severe myalgia, investigations for underlying vitamin-D deficiency or hypothyroidism are recommended, since management of such concomitant disorders has been found to increase the tolerability to statins (Saxon and Eckel, 2016).

Ubiquinone (coQ10) is another factor that might interact with statins and play a role in the reported side effects (Saxon and Eckel, 2016). This coenzyme mediates the aerobic respiration in mitochondria through electron transport (Ernster and Dallner, 1995), and statins have been found to impede its production via inhibition of the mevalonate pathway (Saha and Whayne, 2016; Saxon and Eckel, 2016). In turn, coQ10 deficiency has been reported to correlate with statin-induced myopathy (Choi et al., 2016; Latkovskis et al., 2016; Saha and Whayne, 2016), possibly through ubiquinone-mediated impairment of cellular metabolism (Choi et al., 2016). However, a meta-analysis of RCTs failed to show significant alleviation of statin-associated myalgia by coQ10 supplementation (Banach et al., 2015).

In clinical practice, detailed history and clinical examination are always required when evaluating reported side effects from statin treatment. The type of symptoms, timing, and dose of statin, as well as concomitant treatments and disorders should be recorded. For example, rare, rheumatologic diseases (e.g., giant cell arteritis and polymyositis) may be simultaneously present, hence further investigations (e.g., ESR and CRP) may be required upon clinical suspicion (Saxon and Eckel, 2016). The precise description of muscle symptoms can be helpful in order to establish a probable, possible, or unlikely causal relationship with statins, according to existing scoring systems (Saxon and Eckel, 2016).

In addition to monitoring CK elevations, liver function tests should be also evaluated in statin-treated FH patients (Saxon and Eckel, 2016), including transaminases (aspartate aminotransferase, AST or SGOT, and alanine aminotransferase ALT or SGPT) which are markers of hepatocellular damage (Bolondi et al., 2016). Liver function tests have been found abnormally elevated (in a dose-dependent way) in 0.5–2% of statin-treated patients (Catapano et al., 2016). Although statin-induced elevations in transaminases, especially without parallel bilirubin elevations, are not associated with hepatotoxicity (Herrick et al., 2016), ALT and AST should be assessed, together with CK, at baseline (before the initiation of statin treatment). Then the levels of transaminases should only be reassessed after 8–12 weeks of treatment initiation or increase. Routine follow-up monitoring is currently not recommended. However, if these are elevated, but remain < 3 times the upper reference limit (URL), follow-up tests should be repeated in 4–6 weeks without the need to stop the treatment, whereas levels >3 times the URL require treatment cessation, re-evaluation in 4–6 weeks, and careful re-challenge when normalized (Catapano et al., 2016). Routine CK measurements in asymptomatic patients are also not essential, unless the patient develops myalgia (Catapano et al., 2016). In cases presenting with serious side effects from statin treatment, a referral to a Lipid Specialist physician is required for appropriate discontinuation and potentially a re-challenge strategy (Saxon and Eckel, 2016). Of note, CK cut-offs applied for statin discontinuation differ among specialists and usually local protocols are followed, with Saxon et al. suggesting discontinuation and subsequent repeated renal function tests for CK >10 times the URL (Saxon and Eckel, 2016). The most recent European guidelines for the management of dyslipidemias now recommend the same CK cut-off (>10 × URL) for statin discontinuation, whilst lower CK levels can be considered in case of persistent muscle symptoms (Catapano et al., 2016). Other statin-related side effects appear to include multiple sclerosis, lung disease, hemorrhagic stroke and increased risk of type 2 diabetes mellitus (T2DM) (European Association for Cardiovascular Prevention and Rehabilitation et al., 2011; Barry et al., 2016; Collins et al., 2016). Available data on memory loss in statin-treated elderly patients show neither harm nor benefit, but better designed studies are still required to explore this issue (Samaras et al., 2016). So far, it appears that there is no causal relationship between the spontaneously reported cognitive symptoms and statins (Rojas-Fernandez et al., 2016). Moreover, a systematic review exploring the relation of statins to tendinopathy showed very limited evidence to support the initial hypothesis, whereas particularly simvastatin was strongly correlated with a reduced tendinopathy risk (Teichtahl et al., 2016).

Increased T2DM incidence has been previously reported in clinical trials with statins, but there is some inconsistency in reported findings. Indeed, a study in FH and familial combined hyperlipidemia (FCH) patients failed to show a relationship between high-intensity statin treatment and new onset diabetes (Skoumas et al., 2014). However, a meta-analysis of large RCTs with a minimum 1-year follow-up of statin therapy showed a slight increase in diabetes incidence in hypercholesterolemic patients, mostly treated with atorvastatin and rosuvastatin (Rahal et al., 2016). Underlying diabetes at treatment initiation, intensity of treatment, and lifestyle are considered associated with the diabetes risk in statin-treated patients. To date, the proposed underlying mechanisms for this link involve impaired pancreatic β-cell activity and insulin resistance due to enhanced cellular uptake of cholesterol and pro-inflammatory effects of statins (Ganda, 2016). Additionally, the association of *rs17238484-G* (a genetic polymorphism of the HMG-CoA reductase gene) with increased diabetes risk could potentially offer another explanation for the diabetogenic effect of statins (Swerdlow et al., 2015). Despite these findings, it has been shown that the overall CVD benefit of statins outweighs the diabetes risk in statin-treated patients (Maki et al., 2016).

As the objective of the individualized treatment plan is to keep FH patients on the maximum tolerated statin dose, a series of adjustment maneuvres have been proposed in the literature to better guide the clinical practice. These are outlined in Box 1 and should be applied in close collaboration with the patient. Of note, although these maneuvres seem to be useful in everyday clinical practice, the exact impact on CVD outcomes in patients receiving these modified treatment regimens have not been fully assessed yet, and the available data from clinical trials are not considered sufficient to support an evidence-based consensus (Arca et al., 2012). However, prescribing the maximum tolerated statin dose, with the addition of a non-statin lipid-lowering treatment when indicated, appears to remain the most effective treatment approach in FH patients (Miedema and Virani, 2016). Indeed, as long as even a moderate statin dose is maintained the overall CHD risk can still be significantly reduced (Versmissen et al., 2008). Accordingly, it is crucial that FH patients are informed of the substantial evidence from RCTs showing that the treatment benefits outweigh the risk of side effects in order to reinforce the appropriate use of statins and achieve better clinical outcomes (Collins et al., 2016).

Box 1 **Adjustment maneuvres for treating FH patients with the maximum tolerated statin dose (Tziomalos et al., 2010; European Association for Cardiovascular Prevention and Rehabilitation et al., 2011; Bouhairie and Goldberg, 2015; Saxon and Eckel, 2016).**

**Table:** 

**Adjustment maneuvers for FH patients treated with the maximum tolerated statin dose**
Statin treatment should be stopped if severe symptoms are present. Further discussion with the patient should aim at statin re-challenge when the reported symptoms are alleviated (this approach also allows to evaluate the causative relation to statin use) The same intensity statin group can be maintained; however, on a lower dose (maximum tolerated dose) In case of only mild symptoms, the patient should be offered any statin that hasn't been previously tried (starting with the hydrophilic statins, such as rosuvastatin and pravastatin) Patients on a high-potency statin (e.g., atorvastatin or rosuvastatin) may be switched to an alternate day regimen, or even less often if necessary Statin treatment may be switched to a lower intensity statin group on a nightly or an alternate day regimen with a plan for a slow titration Other lipid-lowering drugs should be added when treatment with only a low statin dose can be maintained and in severe intolerance when statins should be substituted completely

*FH, Familial hypercholesterolemia; CVD, Cardiovascular disease; CAD, Coronary artery disease; LDL, Low density lipoprotein; HDL, High density lipoprotein; Lp(a), lipoprotein (a)*.

### Additional lipid-lowering options in FH

Combination therapy is required in FH patients failing to achieve the desired LDL goals with the maximum tolerated statin treatment (Catapano et al., 2016). The choice of additional lipid-lowering therapy should be based on the assessment of co-existing factors, such as personal history, concomitant medications, the complete lipidemic profile, and risk factors which could precipitate side effects (e.g., myositis; Goldberg et al., 2011). SNPs related to myopathy, like the *SLCO1B1, ABCB1*, and *ABCG2* gene polymorphisms (Stewart, 2013), could be taken into account when considering combined treatment with statins.

#### Cholesterol absorption inhibitors in FH

Ezetimibe selectively blocks the absorption of dietary cholesterol by the intestinal cells and increases cholesterol secretion into the bile at the same time, through interfering with the Niemann-Pick C1-like 1 protein (NPC1L1; Hovingh et al., 2013; McPherson and Hegele, 2015). This leads to reduced intrahepatic cholesterol concentrations and consequent LDLR up-regulation, hence the circulating LDL levels are effectively decreased (Hartgers et al., 2015; Catapano et al., 2016). Interestingly, loss-of-function pathogenic variants of NPC1L1 have not only been associated with reduced LDL levels, but also with a relative 53% decrease in CVD risk, thus drawing more attention as a potential target for CAD management (Myocardial Infarction Genetics Consortium et al., 2014).

Data from clinical trials investigating the ezetimibe effect on LDL show a reduction potential of 15–20%, which is similar either as monotherapy or as an add-on to statin (Hovingh et al., 2013; Hartgers et al., 2015; Catapano et al., 2016). Notably, the combination of statin and ezetimibe appears to induce a significant CVD reduction which is greater than that of statin monotherapy (Cannon et al., 2015; Nussbaumer et al., 2016).

The absence of major side effects, interactions with statins, or restrictions related to liver or renal impairment, renders ezetimibe a relatively flexible treatment option (Catapano et al., 2016). As such, ezetimibe is now considered a valuable weapon in the lipid-lowering arsenal for FH patients, and is recommended as the agent of choice to add on to the maximum tolerated statin dose when LDL targets are not reached, or as monotherapy to statin-intolerant patients (Hartgers et al., 2015; Catapano et al., 2016).

#### Bile acid-binding exchange resins in FH

Cholesterol is partly utilized by hepatocytes to form bile acids, which are secreted to the duodenum and reach the terminal ileum where they are mostly reabsorbed into the enterohepatic circulation. Bile sequestrants disrupt this enterohepatic circulation by combining with bile constituents and preventing their reabsorption. In turn, this leads to increased bile acid excretion via the gastrointestinal (GI) tract and increased utilization of hepatic intracellular cholesterol to form new bile acids (Catapano et al., 2016). In addition, LDLR activity in hepatocytes is also enhanced, resulting in greater LDL absorption and decreased circulating LDL levels (Hovingh et al., 2013; Catapano et al., 2016). Commonly used bile acid exchange resins include colestipol, cholestyramine, and colesevelam, with the latter being a newer drug which appears to have better tolerance, fewer GI side effects and fewer interactions with other medications (Robinson and Keating, 2007; Sonnett et al., 2010; Catapano et al., 2016). Indeed, colesevelam is approved in the US for the treatment of pediatric HeFH patients (10–17 y.o.), with significant beneficial effects on lipid metabolism (Perry, 2010; Lozano et al., 2016).

Overall, clinical trials have shown a potential reduction in LDL plasma levels by 18–25% with resins, with a proportional reduction in CVD (Catapano et al., 2016). Combined with statins, these agents can add up to a 16% greater effect on LDL reduction (Robinson and Keating, 2007). In addition to the expected lipid-lowering effect, colesevelam has been shown to also improve glycemic control (Staels, 2009), potentially acting via suppression of the phosphoenolpyruvate carboxykinase, as well as through enhanced secretion of glucagon-like peptide-1, thus, down-regulating glycogenolysis and increasing insulin secretion (Bays, 2014).

GI symptoms (e.g., flatulence and nausea) are the most common side effects of bile acid sequestrants, which may be alleviated by increased fluid intake. Furthermore, treatment initiation in small increments and slow titration appears effective in controlling these side effects, at least to some extent. In addition, fat-soluble vitamin deficiency may be induced by bile acid sequestrants, as well as increased levels of triglycerides (TG) in predisposed patients. Finally, concomitant ingestion of other drugs should be avoided in order to reduce possible interference with their absorption/metabolism, with the exception of colesevelam [National Cholesterol Education Program (NCEP) Expert Panel on Detection, Evaluation, and Treatment of High Blood Cholesterol in Adults (Adult Treatment Panel III), 2002; Catapano et al., 2016; Turgeon et al., 2016].

In the management of FH, bile acid sequestrants may be recommended either as monotherapy in younger patients, pregnant women or women who want to become pregnant, and patients requiring modest LDL reduction, or as combination therapy with statins in patients with very high LDL levels [National Cholesterol Education Program (NCEP) Expert Panel on Detection, Evaluation, and Treatment of High Blood Cholesterol in Adults (Adult Treatment Panel III), 2002].

#### Nicotinic acid in FH

Nicotinic acid (vitamin B3 or niacin) can be used in patients with hypertriglyceridemia and mixed hyperlipidemias, as it has been reported to increase HDL by 15–35%, whilst decreasing TG by 20–50%, LDL by 5–25%, and Lp(a) by 30% [National Cholesterol Education Program (NCEP) Expert Panel on Detection, Evaluation, and Treatment of High Blood Cholesterol in Adults (Adult Treatment Panel III), 2002; European Association for Cardiovascular Prevention and Rehabilitation et al., 2011]. Nicotinic acid has also been shown to reduce insulin sensitivity and impair glucose control in T2DM patients [National Cholesterol Education Program (NCEP) Expert Panel on Detection, Evaluation, and Treatment of High Blood Cholesterol in Adults (Adult Treatment Panel III), 2002]. However, its effects can be variable [National Cholesterol Education Program (NCEP) Expert Panel on Detection, Evaluation, and Treatment of High Blood Cholesterol in Adults (Adult Treatment Panel III), 2002].

Increased risk of myopathy when combined with statins is a potential side effect of nicotinic acid (Turgeon et al., 2016). Due to neutral CVD outcomes, lack of impact on mortality, and certain side effects (e.g., skin toxicity) reported in two recent trials, nicotinic acid is currently recommended for specific groups of patients that are statin-intolerant, when other therapies have failed to achieve the LDL targets (Najam and Ray, 2015).

#### Fibrates in FH

Fibrates (fenofibrate, bezafibrate, gemfibrozil, ciprofibrate) act as peroxisome proliferator-activated receptor-α (PPAR-α) agonists, resulting in decreased VLDL synthesis and increased TG clearance. In addition, fibrates modestly increase HDL and reduce TC and LDL levels [National Cholesterol Education Program (NCEP) Expert Panel on Detection, Evaluation, and Treatment of High Blood Cholesterol in Adults (Adult Treatment Panel III), 2002; Catapano et al., 2016].

Even though fibrates can reduce CVD events in patients with high TG and low HDL levels (Lee et al., 2011; Catapano et al., 2016), these agents have not been shown to reduce all-cause and CVD-related mortality and morbidity when added to statins (Najam and Ray, 2015). Moreover, the addition of fibrates to statins has been associated with higher incidence of myopathy, rhabdomyolysis and liver dysfunction (Najam and Ray, 2015; Turgeon et al., 2016), and particularly gemfibrozil should not be co-prescribed with statins (Catapano et al., 2016). Thus, fibrates could be considered as an additional option for LDL lowering in HeFH patients, taking into account the increased risk of side effects when added to statins (Najam and Ray, 2015; Turgeon et al., 2016). Fibrates should be generally restricted for treating diabetic patients with HDL < 1 mmol/l (< 40 mg/dl) and LDL of 2.6–3.3 mmol/l (100–129 mg/dl), as add-on to statins/monotherapy, for statin-intolerant patients when LDL is ≥2.6 mmol/l (≥100 mg/dl) (Haffner and American Diabetes, 2004), and for patients with TG >4.5 mmol/l (>170 mg/dl) and low HDL, although high dose statins alone may be also able to achieve at least a moderate effect in these cases (Najam and Ray, 2015).

#### Fish oils in FH

Fish oils have been previously found beneficial in the management of FH, correlating with a less atherogenic lipid profile in FH patients (Friday et al., 1991; Sala-Vila et al., 2013). Moreover, fish oils may be cardio-protective, at least partly, by reducing arterial stiffness and improving blood pressure (Pase et al., 2011; Chan et al., 2016). However, the clinical benefits of fish oil supplementation are not clear, and a recent meta-analysis reported a weakness of fish oils to achieve significant positive outcomes (Grey and Bolland, 2014).

### Plasma exchange in FH

An additional option in the management of FH patients is the mechanical extraction of lipids. Initial trials of plasma exchange in patients with HoFH and HeFH showed significantly reduced cholesterol levels and improved life expectancy (Thompson et al., 1975, 1985; Lupien et al., 1976; Berger et al., 1978).

The apheresis devices work through filtration of the pro-atherogenic lipoproteins [LDL, VLDL, Lp(a)] (Gairin et al., 1990; Moriarty and Hemphill, 2016). It must be noted that, plasma exchange is a non-specific procedure which removes not only LDL, but also albumin, immunoglobulins, coagulation factors, fibrinolytic factors, and HDL. Thus, this particular treatment for FH is considered problematic not only due to the high associated cost, but also because of the increased rate of adverse events and the poor outcomes related mostly to the non-specific HDL removal from the circulation (Health Quality Ontario, 2007). Moreover, difficult venous access in children could be a potential limitation for this treatment (France, 2016).

### Lipoprotein apheresis in FH

Contrary to plasma exchange, lipoprotein apheresis is an expensive, but highly effective procedure which removes LDL (by 50–70%) and Lp(a) from the plasma through an extracorporeal circulation filtering process (European Association for Cardiovascular Prevention and Rehabilitation et al., 2011; Hovingh et al., 2013). Cholesterol is removed by binding to dextran sulfate or heparin molecules and subsequent extracorporeal precipitation (Health Quality Ontario, 2007; Moriarty and Hemphill, 2016). At a low pH, LDL, and Lp(a) are co-precipitated with heparin and the complex is subsequently removed by filtration of the closed loop (Health Quality Ontario, 2007).

In addition to removing cholesterol, heparin-induced extracorporeal LDL precipitation (HELP) protects from atherosclerotic damages by simultaneously filtering fibrinogen and cellular adhesion molecules which also play a role in atherogenesis (Health Quality Ontario, 2007).

The results of the procedure last for up to 2 weeks (Hovingh et al., 2013), hence this is usually provided in weekly or fortnightly intervals (European Association for Cardiovascular Prevention and Rehabilitation et al., 2011), so that the LDL-lowering effects may be maintained in the long-term (Health Quality Ontario, 2007). The long-term benefits of the procedure rely mostly in delaying atherosclerosis, whilst the biological effects can be noted clinically via the regression/resolution of xanthomas (Hovingh et al., 2013).

FH patients failing to reach the desirable LDL levels despite appropriate pharmacotherapy or those with severe atherosclerotic disease should be considered for lipoprotein apheresis in specialized centers (Goldberg et al., 2011). However, it should be taken into account that, lipoprotein apheresis is a time-consuming procedure, which is further associated with increased treatment costs, limited availability, difficulties with venous access, especially for children and certain adverse events (Health Quality Ontario, 2007; Hovingh et al., 2013). The latter include blood loss, hypotension, anemia, chest pain, headaches, flushing, abdominal discomfort, and arrhythmias, with approximate incidence of 1–2% (Hovingh et al., 2013; Moriarty and Hemphill, 2016). Moreover, a systematic analysis has shown that FH patients treated with lipoprotein apheresis may fail to achieve the target LDL goals of < 2.5 mmol/l (< 100 mg/dl), despite the overall reduction effect in LDL plasma levels (Health Quality Ontario, 2007). Nevertheless, lipoprotein apheresis should be considered when the expected benefits exceed the associated risks. Indeed, based on a retrospective study from one center in Germany, long-term lipoprotein apheresis treatment in high risk patients with CVD was shown to be well-tolerated, safe and effective, resulting in decreased LDL and Lp(a) levels and markedly reduced CVD events by 80% during a 6-year follow up period (Heigl et al., 2015). Additional data from centers across Germany showed that lipoprotein apheresis achieved lowering rates exceeding 60% for both LDL and Lp(a) with a 90% decrease in major adverse coronary events (MACE; Schettler et al., 2015).

In clinical practice, lipoprotein apheresis is generally recommended for HoFH or HeFH refractory to diet and drugs (Health Quality Ontario, 2007; Goldberg et al., 2011; Hovingh et al., 2013; Catapano et al., 2016; Moriarty and Hemphill, 2016). The 2011 NLA criteria are outlined and compared to previous guidelines in Table 9. Indeed, due to the nature of HoFH, weekly or biweekly lipoprotein apheresis represents one of the last options in the management of such FH patients, along with maximum doses of high potency statins (Health Quality Ontario, 2007; European Association for Cardiovascular Prevention and Rehabilitation et al., 2011; Hovingh et al., 2013).

**Table 9:** Criteria for lipoprotein apheresis.

**Criteria for LA**	
**1997 US FDA** (≥6 months strictly low in cholesterol diet and maximum tolerated drug therapy) (Health Quality Ontario, 2007)	**2011 NLA** (≥6 months maximum tolerated drug therapy and functional FH) (Goldberg et al., 2011)
HoFH with LDL >13 mmol/l (>500 mg/dl) HeFH with LDL >7.8 mmol/l (>300 mg/dl) HeFH with LDL >5.2 mmol/l (>200 mg/dl) and CAD	HoFH and LDL ≥7.8 mmol/l (≥300 mg/dl), or non-HDL ≥8.5 mmol/l (≥330 mg/dl) HeFH with LDL ≥7.8 mmol/l (≥300 mg/dl), or non-HDL ≥8.5 mmol/l (≥330 mg/dl), and 0–1 risk factors HeFH with LDL ≥5.2 mmol/l (≥200 mg/dl), or non-HDL ≥6 mmol/l (≥230 mg/dl) and ≥2 risk factors, or Lp(a) ≥50 mg/dl HeFH with LDL ≥4.1 mmol/l (≥160 mg/dl), or non-HDL ≥4.9 mmol/l (≥190 mg/dl) and very high risk (established CAD, other CVD, or diabetes)

*LA, Lipoprotein apheresis; FDA, Food and drug administration; NLA, National lipid association; FH, Familial hypercholesterolemia; HoFH, Homozygous familial hypercholesterolemia; HeFH, Heterozygous familial hypercholesterolemia; LDL, Low density lipoprotein; HDL, High density lipoprotein; LP(a), lipoprotein (a); CAD, Coronary artery disease; CVD, Cardiovascular disease*.

### Recent pharmacological options in the treatment of FH

Treatment strategies in the management of FH have investigated the potential role of certain additional options, including microsomal transfer protein inhibitors, liver-selective thyroid hormone mimetics, and oligonucleotides that supress ApoB (mipomersen; European Association for Cardiovascular Prevention and Rehabilitation et al., 2011; Hovingh et al., 2013). Despite promising initial results, additional larger and longer clinical trials are required to establish the exact role of these options in the treatment of FH.

#### Lomitapide

Lomitapide is an oral drug which is approved for the treatment of adults with HoFH (Cuchel et al., 2014a,b; Walsh and Hussain, 2016). Lomitapide acts by inhibiting the microsomal triglyceride transfer protein in the liver, which is necessary for TG absorption by the chylomicrons in the intestine and phospholipids by VLDL in the hepatocytes (Cuchel et al., 2014b). Thus, lomitapide can result in up to 50% reduction of LDL and 15% reduction of Lp(a) levels at 26 weeks of treatment. Of note, its effect on LDL is maintained, although to a lesser degree, from 26 to 78 weeks of treatment, while Lp(a) levels return back to baseline at week 78 (Cuchel et al., 2013). Real-world data from a retrospective study in Italy confirmed the LDL-lowering effect of lomitapide in 15 HoFH patients. Interestingly, follow-up data from this study suggest that 80% of the patients undergoing lipoprotein apheresis could avoid this procedure due to sufficient LDL reduction with lomitapide (D'Erasmo et al., 2017).

Due to common metabolic pathways, co-administration of lomitapide with CYP3A4 inhibitors should be avoided (Cuchel et al., 2014b). Regarding potential side effects, a phase III trial of lomitapide in HoFH patients has shown increased hepatic fat content and elevation of transaminases, which resolved after dose reduction (Cuchel et al., 2013). Hence, monitoring of liver transaminases is necessary during lomitapide treatment (Najam and Ray, 2015). In addition, GI adverse events have been also reported (Cuchel et al., 2013; Stefanutti et al., 2016), which may be addressed with slow dose titration, low-fat diet, and avoidance of meal times (Cuchel et al., 2014b; Roeters van Lennep et al., 2015; Stefanutti et al., 2016). Long-term observational studies, such as the Lomitapide Observational Worldwide Evaluation Registry (LOWER), are still required to better inform the clinical practice on the exact safety and efficacy profile of lomitapide (Blom et al., 2016).

#### Mipomersen

Mipomersen is an antisense oligonucleotide that binds ApoB mRNA and subsequently down-regulates the expression of ApoB by the ribosomes and the production of VLDL (Cuchel et al., 2014b; Najam and Ray, 2015). Mipomersen is administered as a 200 mg subcutaneous once-weekly injection (Hegele et al., 2015); however, it has not been approved for use in Europe yet (Gaudet and Brisson, 2015; Hartgers et al., 2015; Hegele et al., 2015).

Mipomersen is shown to decrease LDL levels by 21% in patients with HoFH and by 28% in HeFH (Hartgers et al., 2015). A phase III randomized, double-blind, placebo-controlled trial studying the addition of mipomersen on the maximum tolerated standard lipid-lowering treatment in HoFH patients showed significant reductions by up to 25, 27, and 31% in LDL, ApoB, and Lp(a) levels, respectively (Raal et al., 2010). Positive lipid-lowering results with mipomersen were also demonstrated in pediatric patients, who were already on the standard of care treatment (Raal et al., 2016a). Moreover, the reductions noted in atherogenic lipoprotein levels with mipomersen correlated with markedly lower incidence of MACE (Duell et al., 2016).

The most common adverse events of mipomersen include transient injection-site reactions and flu-like symptoms, as well as elevated ALT (< 3 × URL in most patients; Akdim et al., 2010; Raal et al., 2010). Mipomersen has also been associated with increased intrahepatic TG content. Indeed, data from an RCT trial showed that 1 in 10 treated patients exhibited reversible (upon discontinuation) hepatic steatosis, while there was also a trend for increased hepatic fat content in the rest of the patients (Visser et al., 2010).

Overall, the use of mipomersen and lomitapide in clinical practice is limited, and these agents are generally prescribed as an add-on to statin treatment in HoFH patients who cannot undergo LDL apheresis (Hartgers et al., 2015).

#### Thyroid mimetics

Thyroid hormones act on two main types of receptors, i.e., thyroid receptors α and β (TRα and TRβ; Villicev et al., 2007; Lammel Lindemann and Webb, 2016). Endogenous thyroid hormones exert lipid-lowering effects through TRβ; however, this effect cannot be utilized for therapeutic purposes due to the concomitant TRα-induced cardiac, muscle and bone thyrotoxic side effects (Lin et al., 2012; Lammel Lindemann and Webb, 2016).

Development of selective TRβ agonists aims to circumvent these problems and could potentially offer an additional approach in FH treatment. Indeed, these thyromimetics can induce hepatic bile acid production and up-regulate the expression of the HDL receptor, i.e., the scavenger receptor type B-Class I (SR-B1), leading to increased transport of cholesterol into HDL particles, even though this effect was not observed in LDLR^−/−^ null mice (Lin et al., 2012). Thus, these agents can interfere with cholesterol metabolism, without the unwanted TRα-related side effects (Villicev et al., 2007). Data from animal studies have shown that treatment of LDLR^−/−^ null mice with selective TRβ agonists, i.e., GC-1 (sobetirome) and KB2115 (eprotirome), decreases serum cholesterol levels by increasing cholesterol utilization for synthesis of bile acids and inducing their subsequent fecal excretion in an LDLR-independent manner (Lin et al., 2012).

Eprotirome administration exhibits a dose-dependent LDL-lowering effect, with evidence from a phase III double-blind RCT in HeFH patients (AKKA trial) indicating that daily oral treatment with a 100 μg dose can result in a 22% LDL reduction compared to placebo after 6 weeks of treatment (Sjouke et al., 2014). However, this study revealed that eprotirome treatment has the potential to induce liver injury (Sjouke et al., 2014), and was prematurely terminated due to other findings of eprotirome-induced cartilage damage in dogs (Sjouke et al., 2014).

Sobetirome administration in various animal studies has resulted in a remarkable LDL reduction, as well as in decreased TG and Lp(a) levels in primates (Lammel Lindemann and Webb, 2016). Despite the absence of thyrotoxicity with the tested doses, this agent appears to be associated with a mild suppression of the hypothalamic-pituitary axis (Lammel Lindemann and Webb, 2016).

Overall, due to the reported side effects, there are doubts about the potential role of selective thyroid receptor agonists as a lipid-lowering therapeutic approach in FH patients. As such, the future role of thyroid mimetics will depend on their safety profile and some of these agents may potentially find a role in HoFH treatment (Lammel Lindemann and Webb, 2016).

#### Proprotein convertase subtilisin/kexin 9 (PCSK9) inhibitors

Proprotein convertase subtilisin/kexin 9 (PCSK9) is a serine protease produced by hepatocytes (Najam and Ray, 2015). PCSK9 blocks the LDLR recycling by mediating clathrin-mediated endocytosis and subsequently inducing the lysosomatic degradation of LDLR (Huff et al., 2014; Najam and Ray, 2015). This leads to LDL accumulation in the circulation (Ferdinand and Nasser, 2015), and eventually promotes atherogenesis, with high PCSK9 levels correlating to the degree of coronary artery calcification (Alonso et al., 2016).

PCSK9 gene pathogenic variants consist of either gain-of-function (leading to high LDL levels), or loss-of-function (associated with a 15–28% reduction in LDL levels and 47–88% reduction in CVD risk) pathogenic variants (Najam and Ray, 2015). The latter rendered PCSK9 inhibitors promising therapeutic measures for FH. In addition to increasing LDL plasma levels, PCSK9 gain-of-function pathogenic variants have been found to increase circulating Lp(a) in FH patients (Tada et al., 2016), potentially by interfering with Lp(a) endocytosis, as shown in a human hepatocellular carcinoma model (Romagnuolo et al., 2015). However, PCSK9 inhibition is not sufficient to restore Lp(a) levels completely, suggesting that additional factors are implicated, such as the up-regulation of ApoB lipoproteins in FH patients (Tada et al., 2016).

To date, PCSK9 has been targeted using a variety of techniques, such as antisense nucleotide therapy and monoclonal antibodies aiming in uninterrupted LDLR expression and, subsequently, substantial clearance of circulating LDL (by up to 70%; Hovingh et al., 2013; Seidah et al., 2014), with reduction of ASCVD-related morbidity and mortality being the ultimate benefit in atherosclerotic and FH patients (Eisen and Giugliano, 2016; Navarese et al., 2016; Sabatine et al., 2016, 2017).

Alirocumab and evolocumab (administrated subcutaneously every 2 and 4 weeks, respectively) represent the two most thoroughly tested PCSK9 inhibitors so far (Gouni-Berthold et al., 2016; Ito and Santos, 2016). A recent meta-analysis of 15 RCTs showed good efficacy and safety data for FH and statin-intolerant patients when administered at least for 8 weeks (Qian et al., 2017). The reported side effects include mild injection-site reactions (Hovingh et al., 2013), upper respiratory tract infections, back pain and influenza (Blom et al., 2014) and very rarely leucocytoclastic vasculitis (Hovingh et al., 2013).

Overall, evolocumab appears to offer an attractive treatment option for both HeFH and HoFH, even in statin-intolerant patients and apart from significantly lowering LDL, it also improves ApoA1, ApoB, Lp(a), non-HDL cholesterol, and triglycerides, ultimately leading to atherosclerotic plaque regression (Wiggins et al., 2018) and may be an alternative solution for FH patients who cannot undergo lipoprotein apheresis. Indeed, a recent study with a small number of participants reported that lipoprotein apheresis showed no superiority on LDL reduction over evolocumab (Lappegard et al., 2016).

Similarly, the ODYSSEY ESCAPE trial demonstrated significant efficacy of alirocumab in 62 HeFH patients undergoing regular lipoprotein apheresis, with 63.4% of the patients escaping lipoprotein apheresis treatment and 92.7% having < 50% of their scheduled sessions (Moriarty et al., 2016). Overall, the ODYSSEY programme of phase III studies with alirocumab has also reported positive outcomes, including evidence in FH patients. This agent has also been well-tolerated, with patients showing good adherence and it has resulted in significant and persistent reductions in LDL, non-HDL cholesterol, and Lp(a) levels (Farnier et al., 2016, 2017; Greig and Deeks, 2016). Pooled data from the Odyssey trials have shown that greater reductions in LDL [including LDL < 1.3 mmol/l (50 mg/dl)], are associated with fewer MACE (Ray et al., 2016). It would be interesting to review the cardiovascular effects of alirocumab when the detailed findings of the ODYSSEY OUTCOMES trial are fully published. Data analysis from 14 trials on alirocumab have documented its safety (Jones et al., 2016). Of note, a systematic review of 12 and 9 phase III trials for alirocumab and evolocumab, respectively, in hypercholesterolemic patients (including FH groups) showed that alirocumab- and evolocumab-treated patients achieved the desirable LDL targets with rates of 87 and 98%, respectively; however, there was no head-to-head comparison between the two drugs (Gouni-Berthold et al., 2016).

Both these PCSK9 inhibitors have been approved by the FDA and the European Medicines Agency (EMA) for HeFH, with evolocumab being also approved for HoFH as additional treatment to diet and maximally tolerated statins for patients at high CVD risk who have not reached their lipid targets, or as replacement to statins when statin treatment is contraindicated or not tolerated (Agabiti Rosei and Salvetti, 2016).

More data on two additional PCSK9 inhibitors, i.e., bococizumab and LY3015014, are being currently collected (Dixon et al., 2016). Indeed, the SPIRE programme enrolling >30,000 patients worldwide, including FH patients, has involved six lipid-lowering and two CVD-outcome studies on the effects of bococizumab (Ridker et al., 2016). Two randomized trials (SPIRE 1 and SPIRE 2) have shown superiority of bococizumab (humanized monoclonal antibody) in MACE only in high risk patients when compared with placebo, however, the trials were discontinued early by the sponsor due to high rates of anti-drug antibodies (Ridker et al., 2017a). Similarly, the rest six parallel, randomized trials with bococizumab have shown a significant reduction in LDL at 12 weeks, however, the results were not sustained for patients with high-titer antidrug antibodies at 52 weeks, whilst patients with no anti-drug antibodies presented wide variation in LDL as well (Ridker et al., 2017b).

As for LY3015014, results so far seem promising with significant reduction in LDL at week 16 and a good safety profile (Kastelein et al., 2016).

Additional results of phase III studies with CVD-outcomes as the primary end point are expected for PCSK9 inhibitors, such as the FOURIER trial which has recently shown a significant reduction on CVD events when adding evolocumab to statin treatment in patients with clinically evident vascular disease (Sabatine et al., 2017).

Due to their satisfying efficacy and safety profile, PCSK9 inhibitors seem to be currently the most promising additional treatment in FH patients who are already on maximum tolerated statin treatment (Reiner, 2015). The 2017 NLA Expert Panel covers FH patients recommending PCSK9 inhibitor treatment for patients with LDL ≥4.9 mmol/l (≥190 mg/dl) and (i) 40–79 y.o., in the absence of poorly controlled ASCVD risk factors and post-treatment LDL ≥2.6 mmol/l (100 mg/dL) or non-HDL ≥3.4 mmol/l (130 mg/dL), while on statin, with or without ezetimibe; (ii) 40–79 y.o., with uncontrolled ASCVD risk factors, or genetic FH confirmation and post-treatment LDL ≥1.8 mmol/l (≥70 mg/dl) or non-HDL ≥2.6 mmol/l (≥100 mg/dl), while on statin, with or without ezetimibe; (iii) 18–39 y.o. with uncontrolled ASCVD or genetic confirmation of FH and post treatment LDL ≥2.6 mmol/l (100 mg/dL) or non-HDL ≥3.4 mmol/l (130 mg/dL), while on statin, with or without ezetimibe; or for (iv) HoFH (unknown genotype/LDLR defective), as additional LDL-lowering therapy, when post-treatment LDL ≥1.8 mmol/l (≥70 mg/dl) or non-HDL ≥2.6 mmol/l (≥100 mg/dl), while on statin, with or without ezetimibe, or before lomitapide, mipomersen and LDL apheresis (Orringer et al., 2017). Table 10 summarizes the recent criteria by **ACC** 2016 (updated in 2017) and ESC/EAS 2017 (updated in 2018), after taking into account the FOURIER outcomes for the general use of PCSK9 inhibitors, including FH patients.

**Table 10:** European and American Criteria for PCSK9 inhibitor use.

**ACC (2016-updated in 2017)**	**ESC/EAS (2017-updated in 2018)**
INDICATIONS: ✓ Either a PCSK9 inhibitor or ezetimibe as a second line agent as an addition to maximum tolerated statin for patients with clinical ASCVD with comorbidities and baseline LDL ≥1.8 mmol/l (70 mg/dL). ✓ Should be preferred when >25% further reduction in LDL is required after discussing all parameters with the patient Specific criteria. Added to statin and ezetimibe: ASCVD without comorbidities and LDL ≥2.6 mmol/L (100 mg/dL) while on maximum tolerated statin and ezetimibe and a reduction of LDL < 50% from baseline ASCVD with comorbidities and LDL ≥1.8 mmol/l (70 mg/dL), or non-HDL ≥2.6 mmol/L (100 mg/dL) in diabetic patients, while on maximum tolerated statin and ezetimibe and a reduction of LDL < 50% from baseline ASCVD with baseline LDL ≥4.9 mmol/L (190 mg/dL) and post-treatment LDL ≥1.8 mmol/l (70 mg/dL) while on maximum tolerated statin and a reduction of LDL < 50%, as an alternative to ezetimibe or bile acid sequestrant without ASCVD and LDL ≥4.9 mmol/L (190 mg/dL) and post-treatment LDL ≥2.6 mmol/l (100 mg/dL) while on maximum tolerated statin and a reduction of LDL < 50%, as an alternative to ezetimibe or bile acid sequestrant before LDL apheresis in HoFH patients, except LDLR negative patients	INDICATIONS: ✓ Adults with HeFH, non-familial hypercholesterolemia, or mixed dyslipidemia with diet, maximum tolerated statin (or when statin-intolerant/contraindicated), or other medications, not achieving LDL goals ✓ Adults and ≥12 y.o. with HoFH on other medications ✓ Symptomatic PAD ✓ Recurrent or recent MI ✓ Multivessel disease Specific criteria: Added on statin and ezetimibe: Severe ASCVD and LDL >2.6 mmol/L (100 mg/dL) ASCVD and LDL >3.6 mmol/L (140 mg/dL) Diabetes with target organ disease or major risk factors (no ASCVD) and LDL >3.6 mmol/L (140 mg/dL) HeFH without ASCVD and LDL >4.5–5 mmol/L (175–200 mg/dL) (according to risk) HoFH (after maximum treatment, including LDL apheresis)—all patients except from those with negative-negative LDLR mutations Statin intolerant patients on ezetimibe and any of the above criteria

*Adapted from Writing et al. (2016), Lloyd-Jones et al. (2017), and Landmesser et al. (2017a,b); Landmesser et al. (2018). ACC, American College of Cardiology; ESC/EAS, European Society of Cardiology/European Atherosclerosis Society; FH, familial hypercholesterolemia; HoFH, homozygous familial hypercholesterolemia; HeFH, heterozygous familial hypercholesterolemia; LDL, low density lipoprotein; HDL, high density lipoprotein; PCSK9, proprotein convertase subtilisin/kexin type 9; ASCVD, atherosclerotic cardiovascular disease; PAD, peripheral artery disease; MI, myocardial infarction*.

However, the high treatment costs (approximate $14,600 annually for alirocumab and $14,100 for evolocumab for HeFH patients) of these agents may pose a barrier for their broader use in routine clinical practice, and it is now known that there should be significant cost reductions to approximately $4,536–$5,459/year/patient to achieve their cost-effectiveness (Kazi et al., 2016; Arrieta et al., 2017). Indeed, a recent Norwegian study has shown that PSCK9 inhibitors are cost-effective only for high-risk older patients during secondary prevention and especially at prices as low as €63,200–68,400 per QALY (instead of the current €81,406–84,646; Korman and Wisloff, 2018). Examples of lifetime costs per patient are for: ezetimibe (€5,000–6,900), PCSK9 inhibitors (€78,000–106,000) (Korman and Wisloff, 2018), and high dose atorvastatin (2005 data of incremental cost-effectiveness ratio—$33,400 per QALY) (Plosker and Lyseng-Williamson, 2007).

#### Cholesterylester transfer protein (CETP) inhibitors

CETP induces the transport of cholesteryl esters and TG from HDL molecules to atherogenic molecules, such as the ApoB-containing lipoproteins (Hovingh et al., 2013). CETP inhibitors include dalcetrapib, torcetrapib, anacetrapib, evacetrapib, and TA-8995, which, apart from increasing HDL, with the exception of the former, appear to decrease LDL plasma levels (Krahenbuhl et al., 2016; Wang et al., 2018). However, despite this effect, recent phase III outcome trials have shown limited benefits on CVD outcomes when these agents are combined with the current standard of care (McLain et al., 2016).

Anacetrapib, not only interferes with lipid exchange, but also reduces LDL by increasing ApoB100-LDL binding to LDLR and removing ApoB from the circulation (Hartgers et al., 2015). As such, the phase III RCT (REALIZE) with anacetrapib as lipid-modifying therapy in HeFH patients demonstrated significant reduction in LDL by 36% after 1 year of treatment with good tolerability (Kastelein et al., 2015). Moreover, the recent REVEAL study has interestingly shown fewer MACE with anacetrapib vs. control in patients with atherosclerotic vascular disease (Group et al., 2017), however, as the greater results were observed much later in years of treatment, this could pose a compliance issue and make it difficult to be successful in real world (Doggrell, 2018).

Torcetrapib has been found to increase HDL by 72.1% and lower LDL by 24.9% in a RCT comparing the combination of torcetrapib and atorvastatin to atorvastatin alone, but this trial was terminated due to increased morbidity and mortality of unspecified pathogenesis (Barter et al., 2007). Moreover, a dalcetrapib trial in patients with recent acute coronary syndrome events showed poor efficacy when added to standard of care treatment, failing to reduce CVD recurrence (Schwartz et al., 2012).

Despite favorable effects on atherogenic lipoprotein-reduction (Nicholls et al., 2016), evacetrapib was also abandoned due to lack of improved CVD outcomes (Filippatos et al., 2016). Finally, despite good results on lipid metabolism with TA-8995, large outcome trials are also needed in order to establish its impact on CVD (Hovingh et al., 2015a; Filippatos et al., 2016; van Capelleveen et al., 2016).

#### Atp-citrate lyase (ACL) inhibitor

The ACL inhibitor ETC-1002 (bempedoic acid) has been found to lower cholesterol biosynthesis by depriving cells of the necessary substrates. To date, studies either as monotherapy or as combination therapy with statins have shown positive results, without significant side effects (Bilen and Ballantyne, 2016; Lammel Lindemann and Webb, 2016). The promising LDL-lowering effects of ETC-1002 from phase II trials remain to be confirmed by a phase III programme assessing its efficacy, safety and long-term outcomes (Bilen and Ballantyne, 2016). Since this agent has been also found to reduce C-reactive protein levels, better CVD outcomes may be possible through its implication in pro-inflammation processes (Penson et al., 2017).

### Plaque regression treatment (rHDL) in FH

Intravenous infusion of reconstituted HDL or a HDL-mimetic particle (CER-001) has shown encouraging results in reversing coronary atherosclerotic damage (Hovingh et al., 2013; Kootte et al., 2015), even in HoFH patients (Hovingh et al., 2015b). CER-001, a pre-beta HDL-mimetic, acts potentially by promoting reverse cholesterol transport and increasing the concentration of ApoA-I. However, sufficient delivery of this agent to atherosclerotic plaques through IV infusion remains a key challenge for this approach (Hovingh et al., 2013; Zheng et al., 2016).

### Surgical therapy in FH

Ileal bypass and liver transplantation could be discussed as treatment options in patients at increased CVD risk who fail to reach the treatment targets or tolerate conventional treatment options (Goldberg et al., 2011; Hovingh et al., 2013; Mansoorian et al., 2015; Martinez et al., 2016).

Liver transplantation is considered a good option for HoFH patients, as the introduction of new functional LDLRs brings the receptor activity close to 60% and reduces LDL plasma levels by 80%. Moreover, by restoring receptor activity, the transplant makes it easier for statins to work on these patients (Bilheimer, 1989).

A recent study in eight pediatric HoFH patients who underwent orthotopic liver transplantation documented an impressive reduction in TC, LDL, Lp(a), and ApoB/ApoAI ratio, which was maintained for 2–6 years. Notably, in the first four of these patients, followed for 4–6 years, CAD did not develop or progress and in fact regressed in two patients with >50% stenosis (Martinez et al., 2016). However, aortic valve stenosis progressed in two of the four patients, while mild hypertension was also reported in two patients (Martinez et al., 2016). Another similar study showed normalization of LDL levels within 1 week after the operation (Alim et al., 2016). However, it must be noted that in order to be curative, the procedure should be performed before CVD is established (Alim et al., 2016; Sanna et al., 2016). Indeed, given the lack of CHD outcome data, more such studies are needed to evaluate the long-term efficacy of this approach (Martinez et al., 2016). Nevertheless, this option should be offered to HoFH patients who lack functional receptors completely in order to maximize the use of the donor capacity (Bilheimer, 1989).

Moreover, a 5-year RCT in hypercholesterolemic patients with previous premature MI (30–64 y.o.) and high cholesterol levels showed positive results in CHD-associated mortality when diet plus partial ileal bypass was compared to diet alone (Buchwald et al., 1998). In addition, the 8-year follow up after partial ileal bypass in three FH patients with xanthomas and CAD showed a 30% reduction in cholesterol and significant change in xanthomas, as well as CAD stabilization (Issa et al., 2000). Thus, partial ileal bypass appears an effective treatment option for FH patients, but more long-term studies are also needed to clarify its safety (Moghadasian et al., 2001).

Portacaval shunt is another surgical option which can reduce the absorption of cholesterol and enhance bile acid excretion (Hovingh et al., 2013). This procedure is known to reduce the rate of TC and LDL synthesis, ultimately leading to significantly reduced plasma LDL levels in HoFH (Bilheimer et al., 1975). Thus, this has been offered as a treatment option to HoFH patients, but also to HeFH patients with severe disease. However, the LDL reduction is approximately 25% in 80% of the treated patients, which is probably not adequate for patients with extremely high LDL levels. As there is significant residual hypercholesterolemia, the portacaval shunt is considered to be a palliative treatment option, while liver transplantation is considered more effective in HoFH (Bilheimer, 1989).

Overall, the existing evidence on the role of the portacaval shunt and ileal bypass in FH treatment remains ambiguous, as the existing literature is quite limited. In 1990, Reeves et al. reported two patients with HoFH treated with the combination of portacaval shunt and mammary coronary bypass grafts, showing good long-term outcomes (Reeves et al., 1990). On the other hand, in a more recent case report two siblings with HoFH who underwent portacaval shunt and ileal bypass were not spared the need for liver transplantation (Lopez-Santamaria et al., 2000).

### Gene-targeted therapy in FH

Genetic therapy may offer a promising approach for the treatment of FH in the near future, since targeting specific genetic loci may lead to precise results with minimal side effects. Indeed, taking into account the effect on LDL clearance of introducing healthy LDLR via liver transplantation, it seems plausible that overexpression of normal LDLR receptors by genetic treatment can achieve similar results (Cuchel et al., 2014b; Najam and Ray, 2015).

Viral vector-associated gene transfer can up-regulate LDLR expression and control hypercholesterolemia in animal models (Kassim et al., 2013; Najam and Ray, 2015). However, a pilot study with retroviral gene transfer to hepatocytes in five HoFH patients resulted in variable biochemical responses, highlighting the need to establish more effective genetic treatment approaches (Grossman et al., 1995).

Recent evidence showed that the inducible degrader of LDLR (IDOL) constitutes a novel LDLR regulator, and prompted the construction of LDLR variants, via specific amino acid substitutions, which were resistant to PSCK9 and IDOL with positive effects on LDL metabolism (Somanathan et al., 2014). IDOL is an E3-ubiquitin ligase which binds to LDLR in a different location than PCSK9, hence promoting receptor ubiquitination and lysosomal degradation (Huff et al., 2014). A study on humanized mice showed that adeno-associated virus-8 (AAV8) mediated expression of IDOL in the liver leads to an LDLR-dependent increase in LDL plasma levels (Ibrahim et al., 2016). As such, further research focused on IDOL inhibitors would be interesting. Indeed, a recent toxicology study assessing the effects of AAV8 expressing directly LDLR in rhesus macaques rendered the treatment safe apart from mild and transient transaminasemia and immune adaptive responses (Greig et al., 2017).

Moreover, AVV-induced RNA silencing methods against ApoB (via short hairpin RNA and artificial microRNA) have led to significant plasma cholesterol reductions (Maczuga et al., 2014). However, substantial changes in the murine liver histology and in genes that are implicated in cell growth, death, immune response, and other basic cell functions have been reported (Maczuga et al., 2014).

The regulation of ApoB splicing represents another approach which could be applied in FH therapy. The post-transcriptional modifications of ApoB appear to be safe and effective in lowering cholesterol by interfering with VLDL assembly and LDL clearance (Khoo, 2015).

Notably, the new era in the development of transgene expression involves advanced recombinant adenoviral vectors which lack viral coding genes, and thus offer genetic therapy without the adaptive immune response and the accompanying chronic toxicity. Gene therapy with helper-dependent adenoviral vectors (HDAd) is an example of this approach (Vetrini and Ng, 2010). HDAd-dependent LDLR gene transport to mice has been proven to be effective against atherosclerosis, despite its moderate LDL-lowering effects (Li et al., 2011). For additional anti-inflammatory effects, PEGylation techniques seem to be useful, while this modification does not interfere with the HDAd vector-induced LDL reduction and atherosclerosis regression (Leggiero et al., 2013).

Rhesus macaque monkeys heterozygous for the mutant LDLR gene (a non-human primate model of FH) have also been tested for HDAd LDLR gene delivery (Oka et al., 2015). This study compared intravenous injection vs. intrahepatic arterial injection in the presence of balloon catheter-based hepatic venous occlusion, and showed that the increased intrahepatic pressure induced by the inhibition of the venous drainage created an optimal environment for gene delivery in the liver. Thus, this method requires lower doses of the viral vectors and maintains the desirable LDL reduction (up to 59%) for a prolonged period (Oka et al., 2015). However, this technique should be further optimized and subsequently tested in clinical studies.

In the pursuit of an effective genetic sequence insertion method, the Sleeping Beauty (transposon) vectors have been also designed (Mikkelsen et al., 2003). These vectors can reach their targets through hydrodynamic gene delivery, since a large volume of fluid (naked DNA solution) is injected into the circulation. Through the effect of hydrostatic pressure, this non-viral technique amplifies endothelial and parenchymal permeability and ultimately achieves genetic material delivery to the desired tissues (Suda and Liu, 2007). Treatment of LDLR-deficient mice with plasmid-based such transposon vectors demonstrated an initial 17–19% decrease in plasma cholesterol which remained stable. This method proved to be safe in mice, offering another potential approach for future trials in FH patients (Turunen et al., 2016).

In order to overcome the host-immune reactions and the technical difficulties of non-viral genetic delivery, Hou et al. recently demonstrated the creation of minicircle non-viral DNA vectors (Hou et al., 2016). After specific modifications and efficient liver-specific LDLR gene expression, correction of hypercholesterolemia in LDLR-deficient mice was reported without significant toxicity, thus offering another potential genetic treatment tool against FH (Hou et al., 2016).

Furthermore, the human induced pluripotent stem cell (hiPSC) technique has shown encouraging results via plasmid vectors (Fattahi et al., 2013). Indeed, transformed differentiated hepatocyte-like cells, either through vectors (Fattahi et al., 2013), or specific genome editing via clustered-regularly-interspaced-short-palindromic-repeats/CRISPR-associated 9 (CRISPR/Cas9) technology, demonstrated increased LDL uptake and correction of the FH phenotype, prompting further investigations (Omer et al., 2017).

Recently, a study in a FH mouse model with a non-viral vector expressing LDLR cDNA combined with a microRNA which suppresses the 3-hydroxy-3-methylglutaryl-coenzyme A reductase (Hmgcr) has also led to a 32% lipid reduction, with a 40% atherosclerotic regression *in vivo* after 12 weeks of treatment (Kerr et al., 2016).

In view of these innovative techniques/approaches, more research efforts have now focused on the development of precise, effective and safe gene delivery strategies for the genetic treatment of FH. These methods seem promising; however, further research is clearly necessary in order to safely induce effective LDLR transgene expression and ultimately achieve sustainable LDL reductions and regression of atherosclerotic disease in humans.

## Conclusions

Presenting with variable genetic, epidemiologic and clinical characteristics, FH is a genetic disease which is increasingly recognized as a significant CVD risk factor that can be effectively managed in everyday clinical practice. Table 11 summarizes the up-to-date management of FH patients, who often remain under-treated, whilst Table 12 further distinguishes the options for HeFH and HoFH, and Box 2 presents the key points in pediatric FH management. Due to the FH-related high CVD morbidity and mortality, early prevention and effective management of these patients is essential through organized primary care and/or Lipid Specialist care centers. Current research further focuses on new monoclonal antibodies/genetic targeting approaches which may offer novel options in order to significantly lower LDL and prevent/reduce ASCVD in FH.

**Table 11:** Summary of management options for familial hypercholesterolemia (FH) patients.

**Treatment option**	**Mechanism of action**	**Effect on lipid metabolism**	**Comments**
Low in cholesterol—saturated fat diet	Reduces cholesterol intake	Up to 10% reduction in LDL	Better CVD outcomes. Must be recommended to all patients along with other lifestyle changes (smoking cessation, alcohol restriction, exercise, blood pressure, and glucose control)
Plant sterols	Affect cholesterol absorption	Not statistically significant results	Cannot be routinely recommended
Statins	Inhibition of HMG-CoA reductase	Up to 80% LDL reduction in HeFH and 20% in HoFH	Important protective impact on CVD outcomes. First line treatment in FH
Ezetimibe	Blocks intestinal cholesterol absorption through the Niemann-Pick C1-like 1 protein	15–20% decrease in LDL	Next add-on drug to statins for even greater CVD protective effect, or as monotherapy for statin-intolerant patients
Bile acid sequestrants	↑ Feacal excretion of bile acids and LDLR up-regulation	18–25% LDL reduction as monotherapy and 16% additional effect with statins	Useful in statin-intolerant patients and pregnant women
Fibrates	PPAR-α agonists (↓VLDL synthesis and ↑TG clearance)	0.4–6% increase in HDL and 15–40% decrease in LDL	Limited use due to side effects, neutral CVD outcomes-restricted mostly to patients with ↑TG and ↓HDL
Niacin	Unclear—↓VLDL synthesis	25% increase in HDL, 20–40% decrease in TG, 15–18% reduction in LDL and 30% reduction in Lp(a)	Limited use due to side effects, neutral CVD outcomes
Fish Oils	Less atherogenic lipid profile (fewer/larger LDL particles, more/larger HDL particles)	20% reduction in TG, 8% decrease in ApoB	Inconsistent findings-recently deemed cardio-protective through increased arterial elasticity results
CETP inhibitors	Inhibit CETP which mediates transport of cholesteryl esters and TG from HDL to ApoB-containing lipoproteins	Up to 25% LDL reduction and 72% HDL increase	Neutral CVD outcomes and increased mortality in some cases
Thyromimetics	Selective TRβ agonists: form bile acids and up-regulate SR-B1	Approximately 22% reduction in LDL	Restricted use due to side effects
PCSK9 inhibitors	Block normal LDLR recycling	LDL reduction: up to a 55% as monotherapy, and 75% combined with a statin	Promising results, but expensive. Good CVD outcomes in recent studies
Lomitapide	Inhibit MTTP which mediates TG and phospholipid absorption	Up to a 50% decrease in LDL and 15% in Lp(a)	Liver and GI side effects. Need for additional safety trials
Mipomersen	Down-regulating ApoB mRNA	Up to 25% LDL and 31% Lp(a) reduction	Limited use due to side effects
Lipoprotein apheresis	Selective mechanical lipid removal	Up to 50–70% LDL reduction	Highly effective, but, due to reasons relating to cost, availability, time-consumption, and adverse events, use is restricted in HoFH or refractory HeFH cases
Liver transplantation	Introduces new functional LDLRs	Up to a 80% LDL reduction	Can be curative if done before established CVD; especially for HoFH
Genetic therapy	Overexpression of normal LDLRs	Results vary according to the applied technique	Promising methods under development

*FH, Familial hypercholesterolemia; HoFH, Homozygous familial hypercholesterolemia; HeFH, Heterozygous familial hypercholesterolemia; LDL, Low density lipoprotein; LP(a), lipoprotein (a); ApoB, Apolipoprotein B; CVD, Cardiovascular disease; LDLR, Low density lipoprotein receptor; TG, Triglycerides; HMG-CoA, 3-hydroxy-3-methyl-glutaryl-coenzyme A; CETP, cholesteryl ester transfer protein; TRβ, Thyroid receptor β; MTTP, Microsomal triglyceride transfer protein; PCSK9, Proprotein convertase subtilisin/kexin type 9*.

**Table 12:** Key points for HoFH and HeFH treatment (Goldberg et al., 2011; Catapano et al., 2016; Jellinger et al., 2017).

**HoFH**	**HeFH**
Prompt diagnosis and early initiation of aggressive treatment Early identification of CAD (especially ostial disease and AS) In addition to lifestyle, statins should be started even in receptor-negative patients Combination therapy usually required (mipomersen, lomitapide, PCSK9 inhibitors) LA necessary in many cases Liver transplantation is an option if available in time Gene therapy seems promising, but needs more clinical trials	Lifestyle changes should precede pharmacotherapy Treatment soon after diagnosis CVD risk factors to be addressed Individualized plan (specific LDL targets agreed with the patient) Statins as first line of treatment Ezetimibe as second line PCSK9 inhibitor could be also an adjunct if eligible Consider polypharmacy side effects

*HoFH, Homozygous familial hypercholesterolemia; HeFH, Heterozygous familial hypercholesterolemia; LDL, Low density lipoprotein; CVD, Cardiovascular disease; CAD, Coronary artery disease; AS, Aortic stenosis; LA, Lipoprotein apheresis; PCSK9, Proprotein convertase subtilisin/kexin type 9*.

Box 2 **Summary of pediatric FH treatment.**

**Table:** 

Mostly similar treatment with adults Increased awareness required Not adequate clinical outcomes data due to enrolment and long-term follow up issues So far, statins ± ezetimibe show promising results regarding LDL and CVD prevention Treatment initiation ≤ 5–10 y.o. in HoFH children Healthy diet and exercise Statin initiation ≥8–10 y.o. LDL targets < 3.5–3.6 mmol/L (< 135–140 mg/dL) for children >10 y.o., or at least a 50% reduction for 8–10 y.o. at very high CVD risk

HoFH, Homozygous familial hypercholesterolemia; LDL, Low density lipoprotein; CVD, Cardiovascular disease. Consensus statements from Wiegman et al. (2015), Catapano et al. (2016), Jellinger et al. (2017), and Harada-Shiba et al. (2018).

## Author contributions

All authors listed have made a substantial, direct and intellectual contribution to the work, and approved it for publication.

### Conflict of interest statement

The authors declare that the research was conducted in the absence of any commercial or financial relationships that could be construed as a potential conflict of interest.

## Abbreviations

ASCVD: Atherosclerotic cardiovascular disease
FH: Familial hypercholesterolemia
HeFH: heterozygous FH
HoFH: homozygous FH
NLA: National lipid association
PCSK9: proprotein convertase subtilisin/kexin type 9 inhibitors
MEDPED: Make early diagnosis to prevent early death
MACE: Major adverse cardiac events.
